# Deep-learning based detection of vessel occlusions on CT-angiography in patients with suspected acute ischemic stroke

**DOI:** 10.1038/s41467-023-40564-8

**Published:** 2023-08-15

**Authors:** Gianluca Brugnara, Michael Baumgartner, Edwin David Scholze, Katerina Deike-Hofmann, Klaus Kades, Jonas Scherer, Stefan Denner, Hagen Meredig, Aditya Rastogi, Mustafa Ahmed Mahmutoglu, Christian Ulfert, Ulf Neuberger, Silvia Schönenberger, Kai Schlamp, Zeynep Bendella, Thomas Pinetz, Carsten Schmeel, Wolfgang Wick, Peter A. Ringleb, Ralf Floca, Markus Möhlenbruch, Alexander Radbruch, Martin Bendszus, Klaus Maier-Hein, Philipp Vollmuth

**Affiliations:** 1 Department of Neuroradiology, Heidelberg University Hospital, Heidelberg, Germany; 2 Division for Computational Neuroimaging, Department of Neuroradiology, Heidelberg University Hospital, Heidelberg, Germany; 3 Division of Medical Image Computing, German Cancer Research Center (DKFZ), Heidelberg, Germany; 4 Helmholtz Imaging, Heidelberg, Germany; 5 Faculty of Mathematics and Computer Science, Heidelberg University, Heidelberg, Germany; 6 Department of Neuroradiology, Bonn University Hospital, Bonn, Germany; 7 Clinical Neuroimaging Group, German Center for Neurodegenerative Diseases, DZNE, Bonn, Germany; 8 Faculty of Medicine, University of Heidelberg, Heidelberg, Germany; 9 Neurology Clinic, Heidelberg University Hospital, Heidelberg, Germany; 10 Department of Diagnostic and Interventional Radiology with Nuclear Medicine, Thoraxklinik at University of Heidelberg, Heidelberg, Germany; 11 Institute for Applied Mathematics, University of Bonn, Bonn, Germany; 12 Heidelberg Institute of Radiation Oncology (HIRO), National Center for Radiation Research in Oncology (NCRO), Heidelberg, Germany; 13 Pattern Analysis and Learning Group, Heidelberg University Hospital, Heidelberg, Germany

**Keywords:** Stroke, Computer science, Diagnostic markers

## Abstract

Swift diagnosis and treatment play a decisive role in the clinical outcome of patients with acute ischemic stroke (AIS), and computer-aided diagnosis (CAD) systems can accelerate the underlying diagnostic processes. Here, we developed an artificial neural network (ANN) which allows automated detection of abnormal vessel findings without any a-priori restrictions and in <2 minutes. Pseudo-prospective external validation was performed in consecutive patients with suspected AIS from 4 different hospitals during a 6-month timeframe and demonstrated high sensitivity (≥87%) and negative predictive value (≥93%). Benchmarking against two CE- and FDA-approved software solutions showed significantly higher performance for our ANN with improvements of 25–45% for sensitivity and 4–11% for NPV (*p* ≤ 0.003 each). We provide an imaging platform (https://stroke.ccibonn.ai/) for online processing of medical imaging data with the developed ANN, including provisions for data crowdsourcing, which will allow continuous refinements and serve as a blueprint to build robust and generalizable AI algorithms.

## Abstract (web summary)

AI may enhance diagnostic accuracy in medicine. Here, authors developed an AI model to detect and localise vessel occlusions in patients with suspected ischemic stroke, outperforming commercial tools on pseudo-prospective multicenter benchmarking.

## Introduction

Imaging constitutes a crucial step in the assessment of patients with acute ischemic stroke (AIS), and CT-based diagnostics offer the quickest and most broadly available solution to demonstrate early ischemic signs, location of the vessel occlusion, and tissue perfusion^1^. The current gold standard of treatment for AIS due to large vessel occlusion (LVO) is the mechanical recanalization of the vessel through angiographic endovascular treatment (EVT), possibly in combination with intravenous thrombolytic agents^2^, and a timely diagnosis of the location and type of vessel occlusion on CT-angiography is critical to reduce onset-to-recanalization time and improve patient outcome^3^. However, a reliable evaluation of the CT-angiography data can be time-consuming, especially for less frequent distal occlusions such as medium-vessel occlusions (MeVOs), which might even be missed by non-experienced readers^4^.

Several commercial computer-aided diagnosis (CAD) tools are available for automated analysis of stroke imaging data, with the goal of enabling neurovascular clinical teams to a faster and more reliable initial diagnosis, improving patient outcomes^5–9^. However, these CAD solutions generally just focus on the detection of large intracranial LVOs of the anterior circulation through an indirect assessment of vascular density on maximum intensity projections, rather than working directly on the original high-resolution data, without the possibility to assess extracranial or posterior circulation occlusions; moreover, they oftentimes present lower performance or even no support for MeVOs or patients with multiple occlusions^10,11^.

In this study, we aimed at developing a deep learning-based tool capable of automatically detecting any type of vessel occlusion from CT-angiography data in the context of AIS, without limiting the analysis to any vessel size or location similarly to a realistic clinical scenario, to expedite the detection of abnormal vessel findings and provide a reliable screening tool to improve clinical workflow in the emergency setting. A large single-institutional retrospective dataset was used for model development, training, and internal testing, whereas multicentre external testing was performed in a pseudo-prospective setting using datasets from three primary/secondary care hospitals of a regional stroke consortium as well as from a tertiary-care university hospital. Furthermore, we benchmarked the performance of our tool in the external data samples against two CE-marked and FDA-cleared software solutions which are currently available on the market.

## Results

**Fig. 1:**
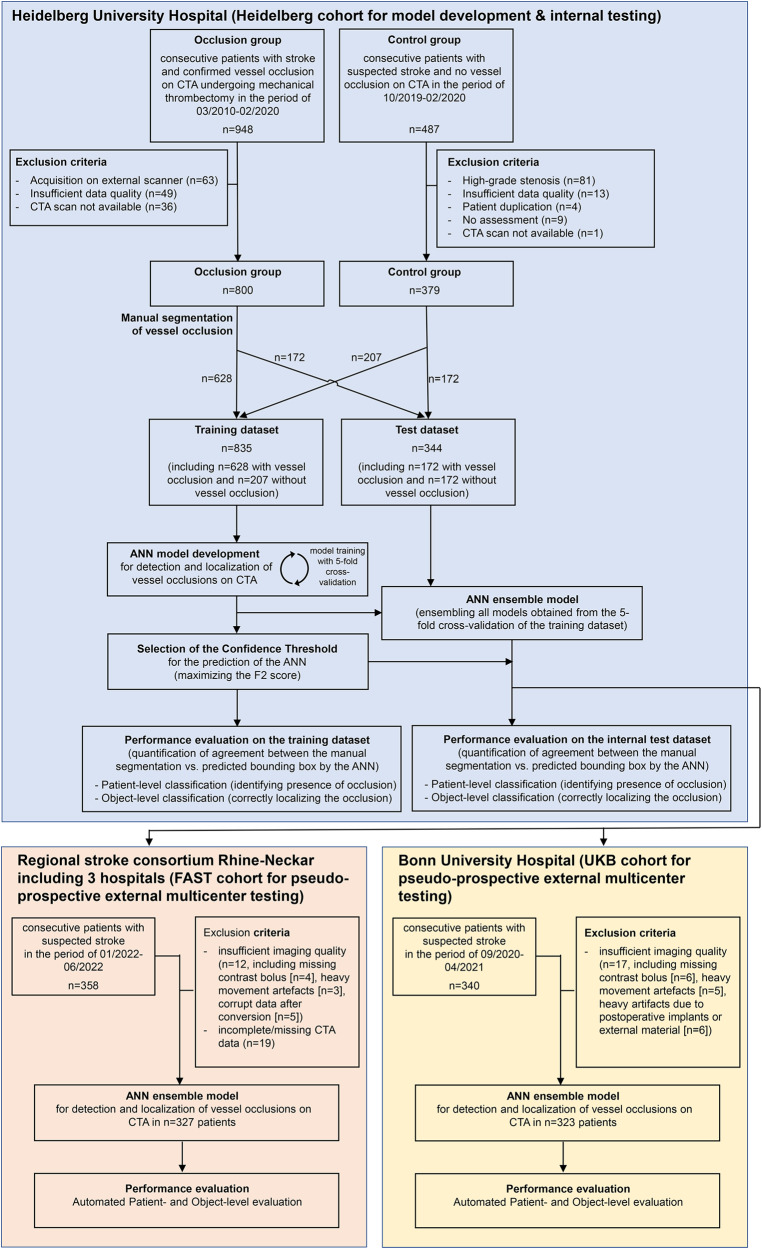
Flowchart of patient selection and procedures. The individual panels for each dataset (Heidelberg, FAST and UKB cohort) summarize patient selection as well as the procedures applied for developing, testing, and performance evaluation of the artificial neural network (ANN) for detecting and localizing vessel occlusions on CT-angiography (CTA).

Detailed information on the distribution of patient demographics, occlusion location and acquisition parameters across the patient cohorts treated at the Heidelberg University Hospital (Heidelberg cohort), at the three primary/secondary care hospitals of the regional stroke consortium Rhine-Neckar with acute teleneurology/teleradiology coverage through the Heidelberg University Hospital (FAST cohort), and at the Bonn University Hospital (UKB) are listed in Supplementary Tables 1–4. A flowchart showing patient inclusion- and exclusion criteria for training and testing of the artificial neural network (ANN) are shown in Fig. 1. The network architecture is depicted in the Supplementary Fig. 1.

The Heidelberg cohort comprised 1179 patients, including 800 consecutive patients (68%) with AIS and either LVO or MeVO who underwent EVT as well as 379 consecutive patients (32%) without vessel occlusion as a control group. Among the 800 patients with AIS, 648 patients (81%) showed one vessel occlusion whereas 152 patients (19%) showed two or more occlusions. A total of 966 vessel occlusions were annotated, including 610 LVOs (63%) and 263 MeVOs (27%) in the anterior circulation and 93 LVOs/MeVOs (10%) in the posterior circulation. Overall, 835/1179 of patients in the Heidelberg cohort (71%) were assigned to the training dataset, including 628/800 patients (79%) with LVO/MeVO and 207/379 control patients (55%) without LVO/MeVO. The remaining 344/1179 patients of the Heidelberg cohort (29%) were assigned to the test dataset, including 172/800 patients (22%) with LVO/MeVO and 172/379 control patients (45%) without LVO/MeVO.

The FAST and UKB datasets comprised 327 and 323 patients with suspected AIS, respectively, and were used for pseudo-prospective testing of the ANN, as described in the following sections. The two datasets presented different scanner models, vendors, slice thickness and acquisition kernels (Supplementary Table 3). Moreover, they also differed in acquisition quality, as demonstrated by the substantially different contrast media distribution phase (Supplementary Table 5); here, the vast majority of patients in both the FAST and UKB dataset presented delayed contrast phases as compared to the Heidelberg dataset, with substantial venous overlay (*p* < 0.001). Patients presented also concurrent intracranial pathology in accordance with an unselected, continuous cohort of patients. For example, patients in the FAST datasets presented *n* = 14 intracranial bleedings, *n* = 2 subdural hematomas, *n* = 8 intracranial aneurysms (*n* = 5 untreated, *n* = 3 post-treatment), *n* = 3 intracranial masses, *n* = 6 intracranial meningeomas. In the UKB dataset, patients presented *n* = 6 aneurysms (*n* = 4 untreated, *n* = 2 post-treatment), *n* = 6 intraparenchymal hemorrhages, *n* = 2 subdural hematomas, *n* = 7 post-operative status after craniotomy (e.g., tumor resection), *n* = 2 AVMs, *n* = 2 intracranial stents.

### Selection of the confidence threshold

First, a confidence threshold for the ANN prediction was determined on the training set of the Heidelberg cohort by maximizing the F2-score, thereby focusing on minimizing false-negatives rather than false-positives (Supplementary Figs. 2 and 3).

### Predictions and inference time measurements

**Fig. 2:**
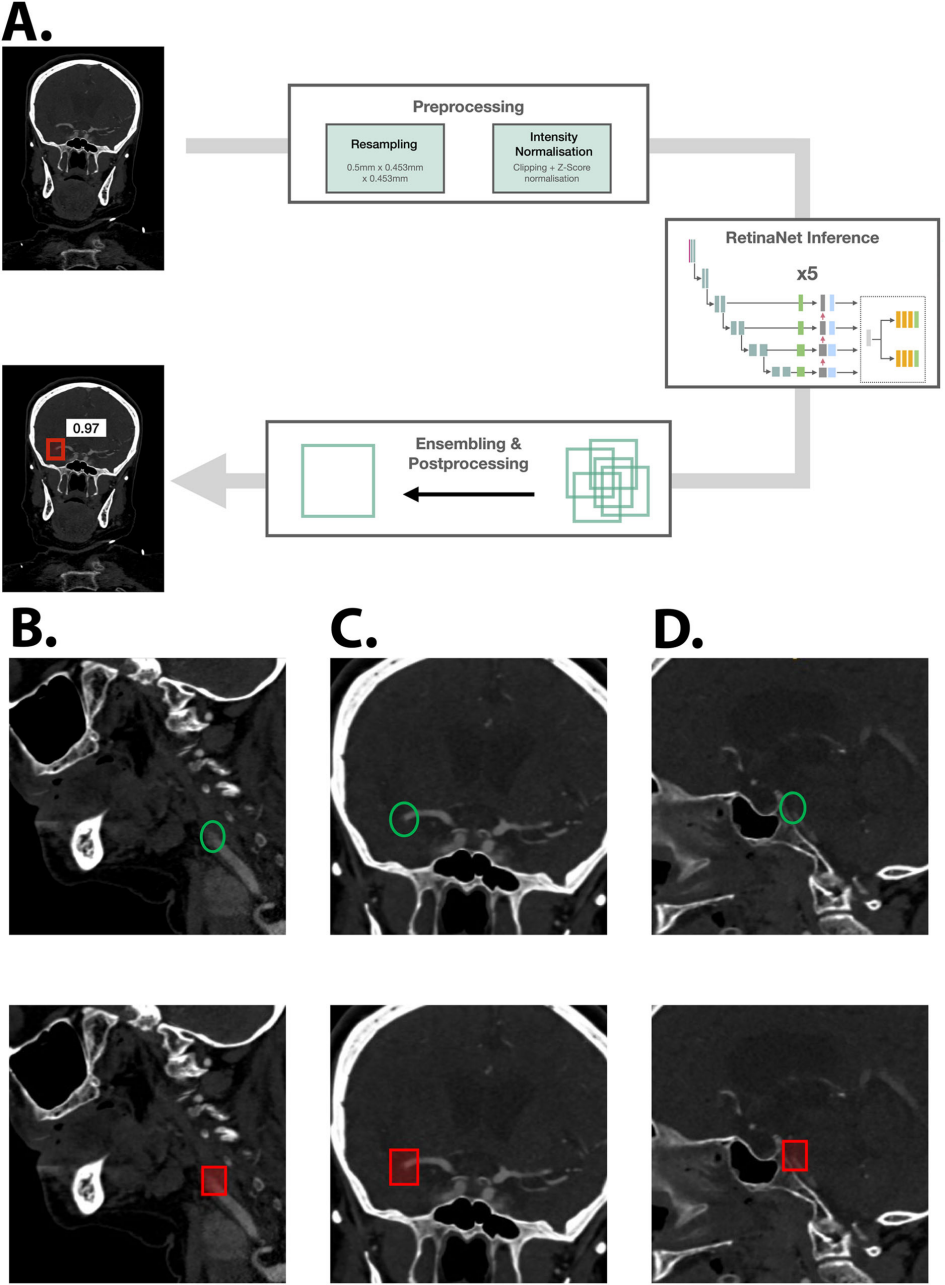
Flowchart of the inference pipeline and example results. The flowchart presenting the inference pipeline of our method is shown in (**A**): data were first pre-processed by resampling the data to the target spacing and normalizing the voxel intensities, and the predictions from the models from fivefold cross-validation are then emsembled into the final label (combination via Weighted Box Cluster). The prediction of the artificial neural network (ANN) is visualized as a red bounding box and the associated confidence score is written next to it. In the lower box, (**B**), (**C**) and (**D**) show representative output samples. The top row shows the underlying ground truth annotation for evaluation as a green circle. The bottom row shows the predicted bounding box by the artificial neural network (ANN) as a red square. **B** Left proximal internal carotid occlusion (sagittal view). **C** Left M1 middle cerebral artery occlusion (coronal view) (**D**) Basilar occlusion (sagittal view). Images are depicted using the radiological standard orientation (left image side corresponds to the patient’s right side).

**Fig. 3:**
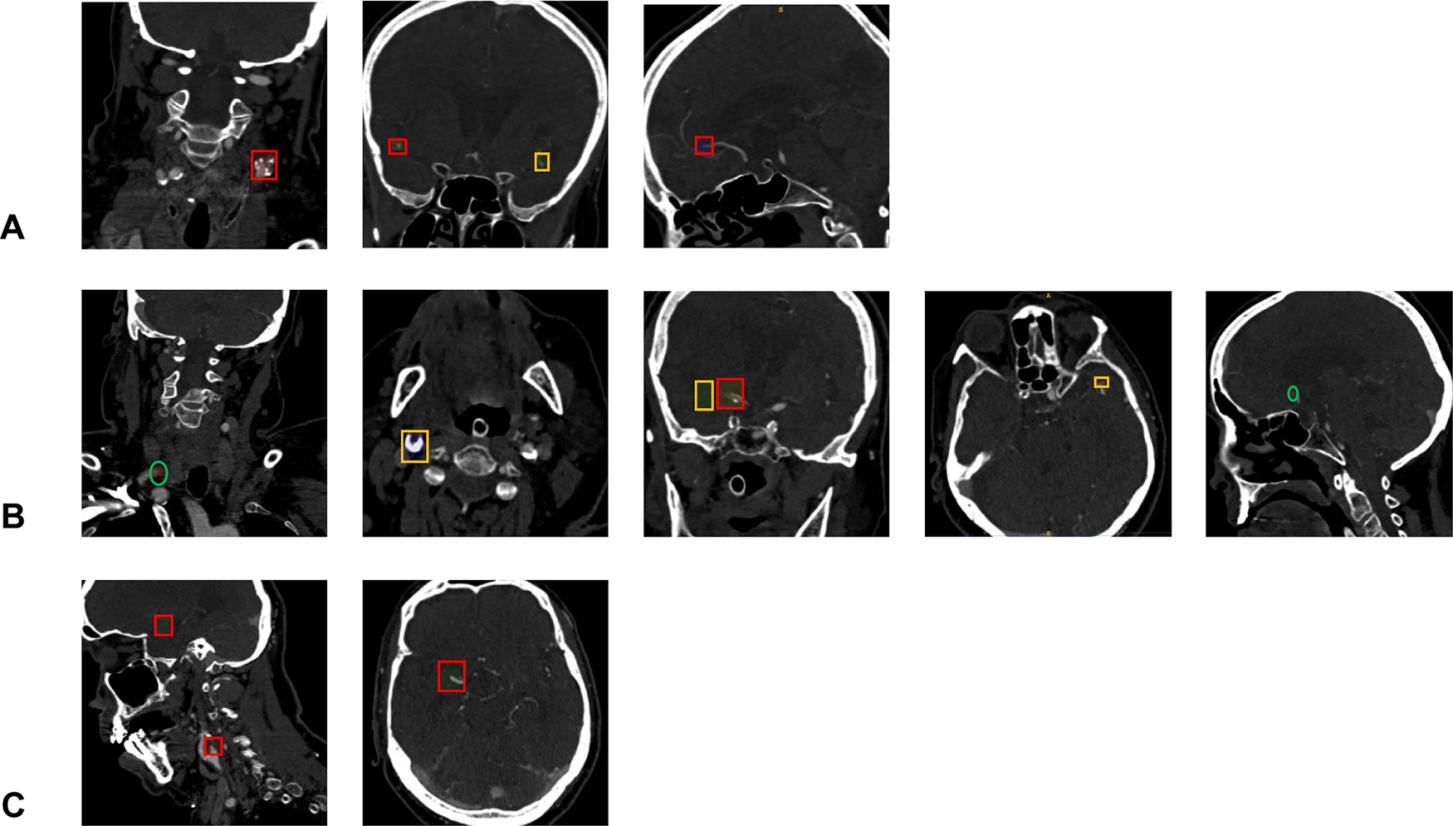
Illustration of prediction results in three patients with multiple occlusions. Correctly predicted bounding boxes by the artificial neural network (ANN) are shown in red, whereas false positive bounding boxes are depicted in orange, whereas for false-negative predictions only the ground truth annotations are shown (green circle). **A** Patient with a correctly ANN- identified occlusion of the left carotid bifurcation (chronic; first slice, coronal), the right middle cerebral artery (MCA) M2-segment (second slice, coronal) and anterior cerebral artery (ACA) A3-segment (third slice, sagittal). In addition, a false positive finding of left MCA M2-segment occlusion was identified by the ANN (second slice, coronal) possibly due to decreased opacification of the vessel due to chronic occlusion of the left internal carotid artery. **B** Patient with occlusion of the right common carotid artery (CCA) not detected by the ANN (first slice, coronal) however false positive prediction of a right carotid bifurcation occlusion distal from the CCA occlusion (second slice, axial). True positive detection of an additional right proximal M1-segment occlusion and false positive of a more distally located M1 occlusion (third slice, coronal). False positive detection of a left MCA M2-segment occlusion (fourth slice, axial), and false negative detection of a right ACA A2-segment occlusion (fifth slice, sagittal). **C** Patient with tandem occlusion of the carotid bifurcation and the proximal MCA M1-segment correctly detected by the ANN (first slice, both occlusion visible, sagittal; second slice - ICA M1-segment occlusion visible, axial). Images are depicted using radiological orientation (left image side is the patient’s right side).

**Fig. 4:**
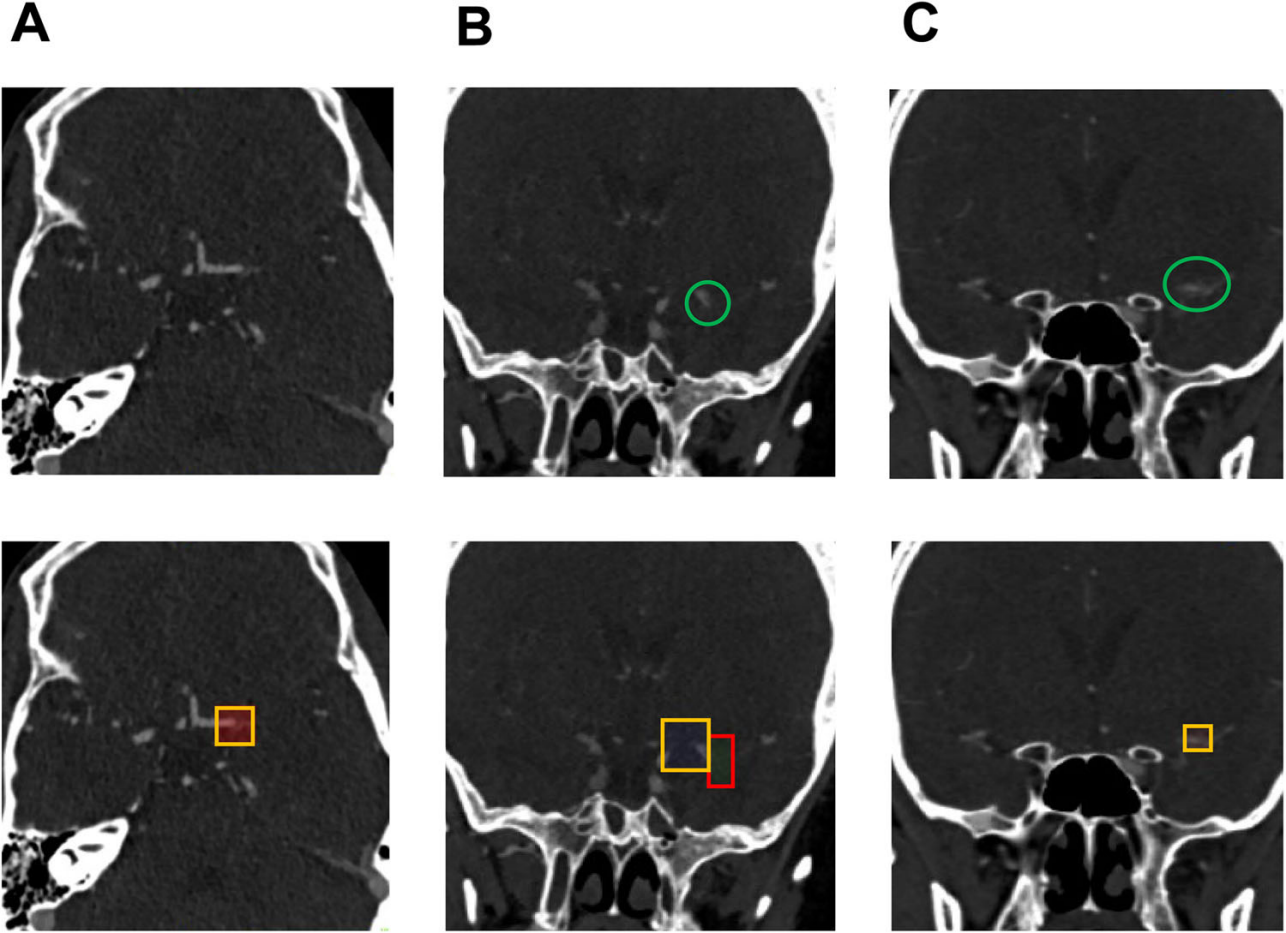
False positive output samples. The top row row shows the underlying ground truth annotation for evaluation as a green circle, if applicable. The bottom row shows the predicted bounding box by the artificial neural network (ANN) as a yellow square for predictions classified as false positive and red square for predictions classified as true positive. **A** False positive prediction at the left carotid T in a patient with proximal internal carotid artery occlusion. The retrograde perfusion of the left A1-anterior cerebral artery is responsible for this impression. (axial view). **B** False positive prediction of a subtotal left M1-middle cerebral artery occlusion, followed by a correctly predicted total left M1 occlusion. (coronal view). **C** M1-middle cerebral artery occlusion classified as false positive. The prediction is correctly located, but the volume of the predicted bounding box is too low compared to the annotation and hence does not reach the intersection-over-union (IoU)-threshold to be classified as true positive. (coronal view). Images are depicted using the radiological standard orientation (left image side corresponds to the patient’s right side).

Illustrative cases with a single occlusion correctly predicted by the ANN are shown with the prediction process in Fig. 2, whereas prediction results from cases with multiple occlusions are shown in Fig. 3; illustrative cases with false-positive predictions by the ANN are shown in Fig. 4.

For each patient in the test set of the Heidelberg cohort, the total processing time, the time used for pre-processing and the time used to predict the ANNs were measured. The total median processing time for a single case was 103 s (IQR 83–142 s), with pre-processing accounting for 83 s (IQR 67–113 s) of processing time and inference of the ANNs accounting for 20 s (IQR 16–28 s) of processing time. Additional details can be found in the Supplementary Fig. 4.

### Patient- and object-level performance—Heidelberg test set

**Fig. 5:**
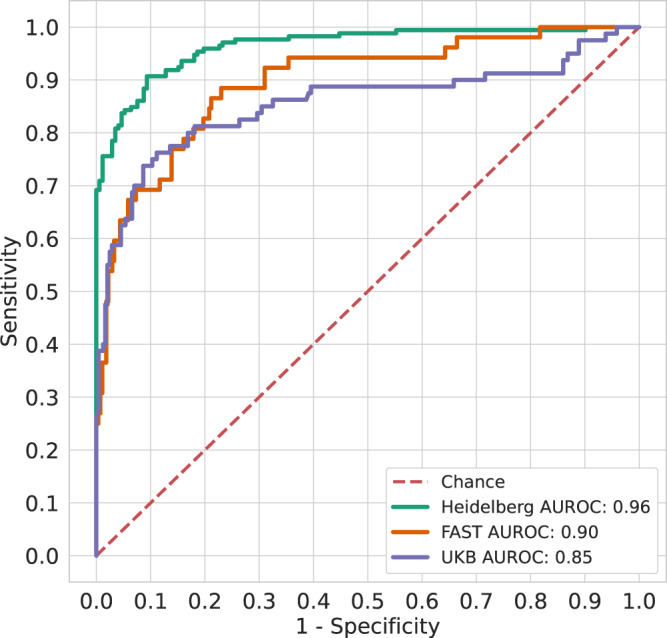
ROCs of the ANN performance in the internal and external test sets. Overall classification performance at the patient level measured by the receiver operating characteristics on the test set of the Heidelberg cohort (green colored), the FAST cohort (orange colored) and the UKB cohort (blue colored). All types of vessel occlusions are considered in the curves (anterior/posterior, LVO/MeVO). Source data are provided as a source data file.

ROC curves for all datasets are shown in Fig. 5. Our method achieved an AUROC of 0.96 (95% CI, 0.95–0.98) for prediction of the presence of a vessel occlusion on CT-angiography within the test dataset of the Heidelberg cohort (see Supplementary Fig. 5). Evaluation at the confidence threshold of 0.647 resulted in a sensitivity of 94% (95% CI, 90–97%), a specificity of 83% (95% CI, 77–88%) and a negative predictive value of 93% (95% CI, 88–96%) on the test dataset (see Supplementary Tables 6, 7 for additional evaluation metrics and the cross-validation cohort).

In terms of object detection our method achieved an overall FROC score of 0.79 (95% CI, 0.73–0.84) for correctly localizing the anatomical site of the vessel occlusion on CT-angiography within the test dataset of the Heidelberg cohort, see Supplementary Tables 8, 9 and Supplementary Fig. 6 for detailed results on the specific occlusion locations and thresholds as well as the cross-validation cohort.

There was a significant association (*R*² = 0.34, *p* = 0.028) between the number of available cases per occlusion site in the training set and the achieved confidence of the prediction for the respective occlusion site in the test set (Supplementary Fig. 7) i.e., higher confidence of the prediction was obtained for those occlusion sites where a greater number of cases were available for training the ANN.

### External testing—FAST and UKB cohorts

The prevalence of vessel occlusions among patients with suspected AIS were 53/327 for FAST (16%) and 85/323 for UKB (26%), including 49/53 patients (93%, FAST) and 76/85 (89%, UKB) with one vessel occlusion and 4/53 patients (7%) and 9/85 (11%) with two or more occlusions, respectively. The distributions of vessel occlusion sites between FAST and UKB was balanced, except for M2-segment occlusions, which were more frequent in the UKB dataset (*n* = 9 vs. *n* = 22 cases, *p* = 0.025; see Supplementary Table 1 for details on the occlusion sites).

The patient-level performance metrics of the ANN in the FAST and UKB cohort as determined by automated, quantitative evaluation (similarly to the Heidelberg dataset) are listed in Tables 1 and 2. Briefly, in the FAST dataset the ANN achieved a patient-level AUROC of 0.90 (95%CI = 0.84–0.94), sensitivity of 0.87 (95% CI, 0.77–0.95), PPV of 0.42 (95%CI = 0.32–0.51) and NPV of 0.97 (95%CI = 0.94–0.99). As listed in Supplementary Table 10 the sensitivity at an object-level was 0.77 (95% CI, 0.62–0.91) for LVOs in the anterior circulation, 0.87 (95% CI, 0.67–1.00) for MeVOs in the anterior circulation, and 0.42 (95% CI, 0.11–0.71) LVOs/MeVOs in the posterior circulation. In the UKB dataset the ANN achieved a patient-level AUROC of 0.85 (95%CI = 0.79–0.91), sensitivity of 0.81 (95% CI, 0.71–0.90), PPV of 0.58 (95%CI = 0.49–0.67) and NPV of 0.93 (95%CI = 0.89–0.96). As listed in Supplementary Table 11 the sensitivity at an object-level was 0.83 (95% CI, 0.70–0.94) for LVOs in the anterior circulation, 0.66 (95% CI, 0.48–0.83) for MeVOs in the anterior circulation, and 0.53 (95% CI, 0.29–0.78) LVOs/MeVOs in the posterior circulation.

**Table 1:** Patient-level performance for detection and localization of vessel occlusions in the FAST cohort

Cohort *n* = Num Patients	AUROC	Sensitivity	Specificity	PPV	NPV
**Full Dataset**					
VO (*n* = 52)/Controls (*n* = 274)	0.90 [0.84, 0.94]	45/52; 0.87 [0.77, 0.95]	212/274; 0.77 [0.72, 0.82]	45/107; 0.42 [0.32, 0.51]	212/219; 0.97 [0.94, 0.99]
VO + HGS (*n* = 79)/Controls (*n* = 247)	0.92 [0.88, 0.96]	69/79; 0.87 [0.79, 0.94]	209/247; 0.85 [0.80, 0.90]	69/107; 0.64 [0.55, 0.74]	209/219; 0.95 [0.92, 0.98]
**Class: VO**					
Anterior Circulation					
LVO (*n* = 23)	0.89 [0.80, 0.97]	20/23; 0.87 [0.73, 1.0]	-	20/82; 0.24 [0.15, 0.34]	212/215; 0.99 [0.97, 1.0]
MeVO (*n* = 14)	0.93 [0.87, 0.97]	14/14; 1.0 [1.0, 1.0]	-	14/76; 0.18 [0.1, 0.27]	212/212; 1.0 [1.0, 1.0]
Posterior Circulation					
VO (*n* = 10)	0.82 [0.65, 0.93]	6/10; 0.60 [0.25, 0.89]	-	6/66: 0.09 [0.03, 0.16]	212/216; 0.98 [0.96, 1.00]
**Class: VO + HGS**					
Anterior Circulation					
LVO (*n* = 32)	0.94 [0.89, 0.98]	30/32; 0.94 [0.85, 1.0]	-	30/68; 0.44 [0.33, 0.56]	209/211; 0.99 [0.98, 1.0]
MeVO (*n* = 20)	0.96 [0.94, 0.98]	20/20; 1.00 [1.0, 1.0]	-	20/58; 0.34 [0.23, 0.46]	209/209; 1.00 [1.0, 1.0]
Posterior Circulation					
VO (*n* = 13)	0.79 [0.65, 0.91]	6/13; 0.46 [0.2, 0.75]	-	6/44; 0.14 [0.04, 0.24]	209/216; 0.97 [0.94, 0.99]

Patients with multiple occlusions were ignored for the automated analysis in the per-class category.

*VO* vessel occlusion, *HGS* high-grade stenosis (>70%).

**Table 2:** Patient-level performance for detection and localization of vessel occlusions in the UKB cohort

Cohort *n* = Num Patients	AUROC	Sensitivity	Specificity	PPV	NPV
**Full Dataset**					
VO (*n* = 80)/Controls (*n* = 243)	0.85 [0.79, 0.91]	65/80; 0.81 [0.71, 0.90]	196/243; 0.81 [0.75, 0.85]	65/112; 0.58 [0.49, 0.67]	196/211; 0.93 [0.89, 0.96]
VO + HGS (*n* = 106)/Controls (*n* = 217)	0.88 [0.83, 0.93]	85/106; 0.80 [0.72, 0.87]	190/217; 0.88 [0.83, 0.92]	85/112; 0.76 [0.68, 0.83]	190/211; 0.90 [0.86, 0.94]
**Class: VO**					
Anterior Circulation					
LVO (*n* = 29)	0.95 [0.88, 0.99]	28/29; 0.97 [0.89, 1.0]	-	28/75; 0.37 [0.27, 0.48]	196/197; 0.99 [0.98, 1.0]
MeVO (*n* = 27)	0.79 [0.66, 0.90]	20/27; 0.74 [0.57, 0.89]	-	20/67; 0.30 [0.20, 0.41]	196/203; 0.97 [0.94, 0.99]
*Posterior Circulation*					
VO (*n* = 15)	0.79 [0.63, 0.92]	10/15; 0.67 [0.40, 0.90]	-	10/57; 0.18 [0.08, 0.28]	196/201; 0.98 [0.95, 0.99]
**Class: VO + HGS**					
Anterior Circulation					
LVO (*n* = 41)	0.95 [0.89, 0.99]	37/41; 0.90 [0.81, 0.98]	-	37/64; 0.58 [0.45, 0.70]	190/194; 0.98 [0.96, 1.00]
MeVO (*n* = 29)	0.85 [0.74, 0.95]	23/29; 0.79 [0.62, 0.94]	-	23/50; 0.46 [0.33, 0.60]	190/196; 0.97 [0.94, 0.99]
Posterior Circulation					
VO (*n* = 20)	0.83 [0.71, 0.93]	13/20; 0.65 [0.43, 0.86]	-	13/40; 0.33 [0.17, 0.47]	190/197; 0.96 [0.94, 0.99]

Patients with multiple occlusions were ignored for the automated analysis in the per-class category.

*VO* vessel occlusion, *HGS* high-grade stenosis (>70%).

### Benchmarking against commercial software products

Benchmarking against two different FDA-cleared and CE-marked software products demonstrated significantly higher performance metrics for the developed ANN in both UKB (Table 3) and FAST cohorts (Supplementary Table 12). To allow for a fair comparison, we limited the benchmarking to the detection of occlusions for which regulatory approval is granted by the two software products. This included the detection of vessel occlusion in the anterior circulation, specifically for occlusions in the internal carotid artery (ICA) and the M1-segment of the middle cerebral artery for LVOs, and in the M2- and M2-segment of the middle cerebral artery for MeVOs.

**Table 3:** Performance benchmarking of two CE-marked and FDA-cleared commercial software (blinded) in the UKB cohort, compared against the developed ANN

UKB DATASET						
Software	AUROC	Accuracy	Sensitivity	Specificity	PPV	NPV
**Overall [Occlusions** ***n* = 65/323]**						
ANN	0.85 [0.80–0.90]	0.86 [0.82–0.90]	0.83 [0.72–0.91]	0.87 [0.82–0.90]	0.61 [0.50–0.71]	0.95 [0.92–0.98]
Commercial Software 1	0.58 [0.52–0.65]	0.70 [0.65–0.75]	0.38 [0.27–0.51]	0.78 [0.73–0.83]	0.30 [0.21–0.42]	0.84 [0.78–0.88]
*p value*	*-*	*-*	**p** < **0.001**	**p** = **0.007**	**p** < **0.001**	**p** < **0.001**
Commercial Software 2	0.65 [0.58–0.71]	0.76 [0.71–0.81]	0.45 [0.32–0.57]	0.84 [0.79–0.88]	0.41 [0.29–0.53]	0.86 [0.81–0.90]
*p value*	*-*	*-*	**p** < **0.001**	p = 0.310	**p** < **0.001**	**p** < **0.001**
**LVO only [ICA, M1 –** ***n* = 38]**						
ANN	0.89 [0.85–0.94]	0.87 [0.83–0.91]	0.92 [0.79–0.98]	0.87 [0.82–0.90]	0.50 [0.38–0.62]	0.99 [0.96–1.00]
Commercial Software 1	0.69 [0.61–0.78]	0.76 [0.–1–0.81]	0.61 [0.43–0.76]	0.78 [0.73–0.83]	0.29 [0.19–0.40]	0.93 [0.89–0.96]
*p value*	*-*	*-*	**p** = **0.001**	**p** = **0.007**	**p** < **0.001**	**p** < **0.001**
Commercial Software 2	0.74 [0.65–0.82]	0.81 [0.76–0.85]	0.63 [0.46–0.78]	0.84 [0.79–0.88]	0.36 [0.25–0.49]	0.94 [0.90–0.97]
*p value*	*-*	*-*	**p** = **0.002**	p = 0.310	**p** = **0.020**	**p** = **0.002**
**MeVO only [M2, M3 –** ***n* = 27]**						
ANN	0.79 [0.69–0.88]	0.85 [0.80–0.89]	0.70 [0.50–0.86]	0.87 [0.82–0.90]	0.35 [0.23–0.49]	0.97 [0.93–0.99]
Commercial Software 1	0.43 [0.37–0.48]	0.71 [0.66–0.77]	0.07 [0.01–0.24]	0.78 [0.73–0.83]	0.03 [0.00–0.12]	0.89 [0.84–0.93]
*p value*	*-*	*-*	**p** < **0.001**	**p** = **0.007**	**p** < **0.001**	**p** < **0.001**
Commercial Software 2	0.51 [0.43–0.59]	0.78 [0.72–0.82]	0.19 [0.06–0.38]	0.84 [0.79–0.88]	0.11 [0.04–0.23]	0.91 [0.86–0.94]
*p value*	*-*	*-*	**p** < **0.001**	p = 0.310	**P** = **0.002**	**p** < **0.001**

Comparisons were performed visually for all patients by considering only the detection of occlusions of the anterior circulation. Findings were considered correct as long as labeled on the correct vessel, in order to provide a fair comparison between the software. Findings labeled in vascular territories not considered by the commercial software (e.g., posterior circulation) were ignored.

McNemar’s two-tailed test was used to compare specificity and sensitivity; comparison of relative predictive values was used instead to compare PPV and NPV (rpv.test function of R’s DTComPair package, two-tailed). *P* values refer to the comparison between the developed ANN and the respective commercial software, and are reported without correction for multiple comparisons. *P* values considered significant are highlighted in bold.

Specifically, for the UKB cohort the developed ANN achieved a patient-level sensitivity of 0.83 (95%CI = 0.72–0.91), which was higher as compared to Software 1 (subsequently referred to as S1) with 0.38 (95%CI = 0.27–0.51), and as compared to Software 2 (subsequently referred to as S2), with 0.45 (95%CI = 0.32–0.57) (*p* < 0.001 each); it also achieved a higher PPV of 0.61 (95%CI = 0.50–0.71) vs. 0.30 (95%CI = 0.21–0.42) for S1 and 0.41 (95%CI = 0.29–0.53) for S2 (*p* < 0.001 each), and NPV of 0.95 (95%CI = 0.92–0.98) vs. 0.84 (95%CI = 0.78–0.88) for S1 and 0.86 (95%CI = 0.81–0.90) for S2 (*p* < 0.001 each). The same results were maintained when examining the performance for LVO- (ICA and M1-segment occlusions) or MeVO-only (M2- and M3-segment occlusions). Again, the developed ANN achieved a higher sensitivity (0.92 [95%CI = 0.79–0.98] vs. 0.61 [95%CI = 0.43–0.76] for S1 vs. 0.63 [95%CI = 0.46–0.78] for S2, *p* < 0.001 each), a higher PPV (0.50 [95%CI = 0.38–0.62] vs. 0.29 [95%CI = 0.19–0.40] for S1 vs. 0.36 [95%CI = 0.25–0.49] for S2, *p* < 0.001 each) and a higher NPV (0.99 [95%CI = 0.96–1.00] vs. 0.93 [95%CI = 0.89–0.96] for S1 vs. 0.94 [95%CI = 0.90–0.97] for S2, *p* < 0.001 each) for LVOs, and maintained a higher sensitivity (0.70 [95%CI = 0.50–0.86] vs. 0.07 [95%CI = 0.01–0.24] for S1 vs. 0.19 [95%CI = 0.06–0.38] for S2, *p* < 0.001 each), a higher PPV (0.35 [95%CI = 0.23–0.49] vs. 0.03 [95%CI = 0.00–0.12] for S1 vs. 0.11 [95%CI = 0.04–0.23] for S2, *p* < 0.001 each) and a higher NPV (0.97 [95%CI = 0.93–0.99] vs. 0.89 [95%CI = 0.84–0.93] for S1 vs. 0.91 [95%CI = 0.86–0.94] for S2, *p* < 0.001 each) for MeVOs. Table 3 includes a complete list of performance metrics and confidence intervals.

Due to contractual limitations, benchmarking for S1 was not possible in the FAST cohort. Performance of S2 as compared to the developed ANN in the FAST cohort is listed in the Supplementary Table 12. Here, the developed ANN again demonstrated again a higher sensitivity of 0.92 (95%CI = 0.79–0.98) vs. 0.67 (95%CI = 0.50–0.81) for S2 (*p* = 0.003), as well as a higher PPV of 0.46 (95%CI = 0.35–0.58) vs. 0.34 (95%CI = 0.23–0.45) for S2 (*p* = 0.021) and a higher NPV of 0.99 (95%CI = 0.96–0.1.00) vs. 0.95 (95%CI = 0.91–0.97) for S2 (*p* = 0.003).

### Inclusion of high-grade stenosis

For the subset analysis including HGS besides vessel occlusions as prediction target—as referenced in the methods, 40 HGS were recorded among the 327 patients of the FAST dataset, including 20 on large vessels (40%) and 14 on medium-sized vessels (35%) in the anterior circulation, and 6 within the posterior circulation (15%). In the 323 patients of the UKB dataset we observed 36 HGS, including 20 on large vessels (55%) and 7 on medium vessels (19%) in the anterior circulation, and 9 in the posterior circulation (25%). A total of 16/40 HGS were located at the bifurcation of the carotid artery in the FAST dataset (40%) and 15/36 in the UKB dataset (42%).

For this task the ANN demonstrated in the FAST cohort a patient-level AUROC of 0.92 (95%CI = 0.88–0.96), sensitivity of 0.87 (95% CI, 0.79–0.94), PPV of 0.64 (95%CI = 0.55–0.74) and NPV of 0.95 (95%CI = 0.92–0.98). In the UKB dataset, the ANN showed an AUROC of 0.88 (95%CI = 0.83–0.93), sensitivity of 0.80 (95% CI, 0.72–0.87), PPV of 0.76 (95%CI = 0.68–0.83) and NPV of 0.90 (95%CI = 0.86–0.94). The corresponding object-level performance are listed in Supplementary Tables 10 and 11.

### Confidence scores and false positives

**Fig. 6:**
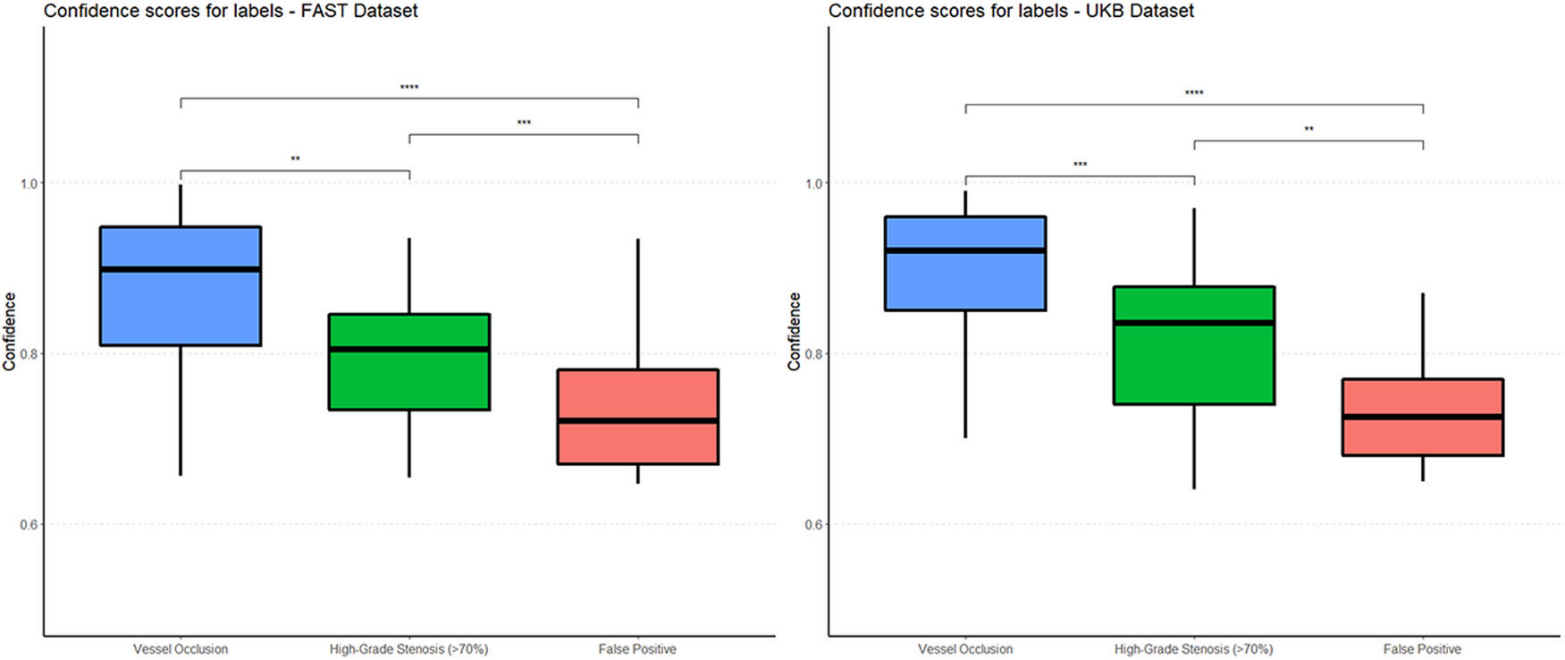
Differences in confidence scores for predictions and false positives. Plotted confidence scores for the vessel pathology detected by the ANN in each of the observed categories within the FAST and UKB dataset, demonstrating the utility of the confidence scores for prioritization of findings within a simulated clinical setting. Groups are compared using the two-sided Wilcoxon rank-sum test. Statistical significance is highlighted between the groups. In both datasets the ANN demonstrated significantly higher confidence when detecting vessel occlusions (VO, depicted in blue color on the left, median FAST (*n* = 47) 0.90 [0.81–0.95] and UKB (*n* = 71) 0.92 [0.85–0.96]) as compared to high-grade stenosis (HGS, depicted in green color in the middle, median FAST (*n* = 32) 0.81 [0.74–0.85], *p* = 0.002 and UKB (*n* = 22) 0.84 [0.74–0.88], *p* < 0.001), as well as compared to false positive results (FP, depicted in salmon color on the right, median FAST (*n* = 48) 0.72 [0.67–0.78], *p* < 0.001 and UKB (*n* = 44) 0.73 [0.68–0.77], *p* < 0.001). The ANN demonstrated also higher confidence when detecting HGS as compared to FP (FAST, *p* < 0.001 and UKB, *p* = 0.001). Data boxes depict median values (center) ± interquartile range (bounds of box), bars depict data range. *P* values are reported without correction for multiple comparisons.

In the FAST and UKB dataset, the ANN demonstrated significantly higher confidence scores when detecting true-positive vessel occlusions (VO, median FAST 0.90 [0.81–0.95] and UKB 0.92 [0.85–0.96]) as compared to HGS (median FAST 0.81 [0.74–0.85], *p* < 0.05 and UKB 0.84 [0.74–0.88], *p* < 0.001), as well as compared to false positive results (FP, median FAST 0.72 [0.67–0.78], *p* < 0.05 and UKB 0.73 [0.68–0.77], *p* < 0.05). The developed ANN demonstrated also higher confidence when detecting HGS as compared to FP (*p* < 0.001). (Fig. 6). There were 48 false positive findings of the ANN in the FAST dataset and 44 in the UKB dataset, and the majority of them (26/48 [54%] and 27/44 [61%]) were located on small veins rather than arterial vessels (Supplementary Table 13).

### Public web platform

Finally, the developed ANN was deployed to an online platform (https://stroke.ccibonn.ai/) which allows online processing of CT-angiography data (for research purposes only). The platform was implemented using Kaapana (https://github.com/kaapana/kaapana), which is an open source toolkit for state of the art platform provisioning in the field of medical data analysis^12^. Specifically, the web platform allows to upload CT-angiography data in DICOM format, with subsequent execution of an automated workflow which includes (1) automated de-identification routines (including de-facing) for the uploaded data, (2) image pre-processing, (3) inference by the ANN and (4) image post-processing, according to the specifications listed in the Methods and Supplementary Methods. The website then provides prediction results (presence and localization of vessel occlusions) through an embedded web-based DICOM viewer. Moreover, the platform includes provisions for data crowdsourcing (i.e., optional donation of CT-angiography data after upload), which may serve as a basis for continuous refinements of our ANN and as a blueprint to build robust and generalizable AI algorithms.

## Discussion

In this study we developed an ANN capable of automatically labeling vessel occlusions in the context of AIS without any a-priori restrictions and irrespectively of number of occlusions, anatomical location or vessel size, which also worked directly on high-resolution CT-angiography data without extensive pre-processing, and therefore resembled the real-life evaluation performed by radiologists. The ANN was developed and tested onto a large single-institutional dataset, and was capable of predicting the presence of a vessel occlusion on CT-angiography data in less than 2 min, thus enabling a possible integration for the triage of CT scans in the emergency setting. External testing was performed in a pseudo-prospective setting with continuous data from all patients which received CT-angiography for suspected AIS within a 6-month timeframe at three different primary/secondary care hospitals from a regional stroke consortium and at a tertiary-care university hospital, with data representing a heterogeneous real-world setting. Specifically, this included CT data from different vendors, scanner models, acquisition and reconstruction protocols, and contrast phases. Here the developed ANN confirmed its high NPV, which was our key metric by design choice and indeed one of the most crucial in an emergency setting: while positively labeled scans will inevitably undergo rapid assessment, missed vessel occlusion might result in acute findings receiving a wrongfully lower prioritization, further increasing imaging-to-treatment times. The developed ANN demonstrated object-level performance that reflected the distribution of occlusion site frequencies in the training set, and the external analysis also highlighted the importance of confidence scores for guiding the interpretation of the ANN’s decisions in clinical practice, as the ANN produced high confidence scores when correctly identifying occlusions but low confidence scores when producing false positives. As a secondary analysis, the ANN showed stable performance also with the inclusion of HGS within the external datasets; although this was not the primary purpose of the current study, this finding was still useful when considering a potential clinical application and demonstrated the potential for future expansions of the ANN to other domains of vessel pathology through new, targeted training datasets. When benchmarked against two CE-marked and FDA-approved software solutions which are currently on the market, the developed ANN demonstrated significantly higher performance metrics. Specifically, the developed ANN yielded a 25–45% increase for sensitivity and a 4–11% for NPV (*p* ≤ 0.003 each) for the subset of occlusions for which regulatory approval is granted by the two software products, with the largest performance gaps observed for cases with MeVOs (here defined as M2- and M3-segment occlusions).

Radiology departments are facing an increasing workload^13^, often resulting in staff shortages as well as an overload of the workforce in many western countries. In the context of AIS, detection of vessel occlusions can be a demanding task for non-specialized personnel, resulting in increased error rates^4^, and multiple commercial solutions have been developed for automated AI-based detection of vessel occlusions. For the detection of LVOs using available commercial software, a sensitivity of 96% and specificity of 98% has been reported for RAPID-LVO (RapidAI), 82% and 90% for Viz-LVO (Viz.ai), 84% and 96% for e-CTA (Brainomix), 93–96% and 78% for StrokeViewer (NicoLAB) as well as 73% and 98% for ^AUTO^Stroke LVO (Canon)^5–9^. However, these solutions are limited by primarily focusing on the detection of anterior circulation LVOs, while the performance for (potentially also endovascularly treatable) MeVOs in the anterior circulation is substantially lower (e.g., with reported sensitivities of 49–72% for detecting M2 occlusions^8,9^ using StrokeView, ^AUTO^Stroke LVO or RAPID-LVO)^6,14^. Compared to these studies, the developed ANN demonstrated stable, high performance also when assessing MeVOs, and provided support for posterior circulation and extracranial occlusions. Moreover, the ANN was successful in maximizing NPV, our target metric, demonstrating scores ≥0.93 across all datasets, despite differences in scanner hardware, acquisition protocols and contrast phases. Assessment of two different CE-marked and FDA-approved software solutions showed significantly lower performance (as compared to the developed ANN) for both LVOs and MeVOs. The performance metrics of the two evaluated software solutions were also well below the reported values in the literature, most likely reflecting the challenging real-world setting of our study, which included all consecutive patients with suspected stroke from a 6-month time period from four different hospitals for external testing, therefore also including different scanner hardware and acquisition protocols (e.g., CT-angiography data with venous overlay, which adds an additional layer of complexity). Taken together these results may provide a more realistic performance assessment of commercial software products for this task and may allow to address potential selection biases of previous studies.

Although all of the commercial solutions previously mentioned are based on a convolutional neural network (CNN) algorithm to detect LVOs, each vendor uses different modifications of this method, and may prefer high sensitivity at the expense of specificity or vice versa. As far as disclosed, and also in the case of the two benchmarked software solutions, available tools seem to use CNN-based approaches for identifying vessel asymmetries (as compared to the contralateral hemisphere) that are suggestive of an LVO and thereby aim to identify the presence (LVO yes vs. no) and also infer the anatomical localization of the vessel occlusion^15^. While providing an easy solution for larger vessel occlusion, this approach could at least partially explain the lower performance for smaller vessel occlusions in peripheral vascular territories (e.g., M2 segment), where a dramatic drop in vascular density is at times not present or presents significant interindividual variability due to the different possible anatomical configurations of the M2-subdivision (e.g., bifurcation vs. trifurcation)^7,10,11,16^. Moreover, this method could also fail to appreciate the large inter-personal differences of vascular symmetry which could occur after a vessel occlusions due to different levels of compensatory vascularization through distal collaterals. Overall, the large performance gap between the benchmarked software and the developed ANN suggests that a reconstruction-independent approach to the detection of vascular occlusions in AIS could be superior to existing (commercial) methods, especially in the case of smaller vessels.

Although intracranial LVOs in the anterior circulation constitute the most frequent type of occlusions (ranging from 39 to 83% in the previous literature^17,18^), they are also intuitively those with the quickest learning curve for non-specialized radiologists and ER personnel, and the detection of occlusions in infrequent locations or throughout smaller vessels (even in the anterior circulation) is a more demanding and time-consuming task. In this context, available commercial CAD tools at times fail in supporting the most difficult diagnostic segments, and none of them have yet focused on the detection and localization of (potentially also endovascularly treatable) LVOs or MeVOs in the posterior circulation.

We found encouraging performance on unseen, external data in both the FAST and UKB cohorts, with very high NPV across all institutions. Additionally, we also performed a subset analysis by including the detection of HGS as prediction target besides vessel occlusions, because we noticed during the review process that HGS were also labeled by the ANN. Despite being trained purely to detect occlusions, we found stable or even improved performance of the ANN when also including HGS, although these were labeled with comparatively lower confidence. Overall, this suggests that a steep reduction of vessel caliber might be sufficient to induce the developed ANN in producing a pathological label, even without a complete stoppage of the arterial opacification in the following segments. From a clinical perspective, this unexpected behavior of the ANN might be of added value: high-grade stenoses are a clinically relevant entity when evaluating patients with stroke symptomatic, which in the acute setting might even require endovascular or surgical treatment if producing symptomatic perfusion deficits despite optimal blood pressure control. However, further targeted testing and/or re-training of the developed ANN on larger samples might be warranted to fully address this pathology.

Upon review of the acquisition phase of the CT-angiography data we also noticed a systematic shift towards delayed acquisition phases in the FAST and UKB cohorts as compared to the Heidelberg cohort. This led to a higher frequency of venous overlay, and during the assessment of false positive findings by the ANN in the FAST and UKB cohort we indeed noticed that the majority of them were located on small venous vessels rather than on arteries. Here, the partial opacification of the venous system at delayed acquisition phases might have confused the developed ANN by highlighting small or medium veins which then seemingly disappeared into the brain tissue, thus mimicking the appearance of a vessel occlusion. Suboptimal contrast phase complicates the evaluation of CT-angiography in the clinical setting, and similarly as to how human readers require increased effort to evaluate these exams, the ANN was also confused by the venous overlay, producing low-confidence false positives on venous vessels. This further highlights the importance of consistent, high-quality acquisition schemes in the early arterial phase for CT-angiography, especially when applying CAD systems, to avoid higher false positive rates.

Our study has some limitations. First, while we tried to collect a large dataset, there was a clear class imbalance for some of the less common vessel occlusions which affected the object-level performance of the ANN for under-represented occlusions. Although the NPV—which was excellent throughout the whole cohort, even for underrepresented occlusion sites – may be regarded as the key metric when evaluating the screening capabilities of the method, future large-scale studies focusing on novel decentralized AI algorithms, as well as our publicly available crowdsourcing platform (http://stroke.neuroAI-HD.org) may foster multi-site collaborations and allow to further improve the object-level performance for less common vessel occlusion sites, specifically in the posterior circulation. Second, although we found encouraging results regarding the detection of HGS besides vessel occlusions, it requires further validation and/or re-training on targeted populations to prove its clinical efficacy. Third, although the current median processing time of 103 s is already largely within an acceptable clinical timeframe, it may be further reduced by improving the pre-processing time for each patient, which currently accounts for more than 80% of the total processing time for each case in our study. As we relied on commonly available freeware software packages, the pre-processing in our study was entirely performed through the central processing unit (CPU) due to the lack of Graphics Processing Units (GPU)-accelerated libraries for some required operations. Future studies should therefore focus on implementing a fully GPU-based prediction pipeline, which would allow for faster resampling and to remove additional time spent to transfer information between different computing units. Fourth, although benchmarking was performed using two widely used CE-marked and FDA-approved software solutions for identifying vessel occlusions on CT-angiography in patients with suspected acute ischemic stroke, potentially different (higher) performance metrics may have been achieved with other commercially available software solutions that were not evaluated within this study. Finally, our tool does not currently perform an automated quality control for the input data, and further developments should aim at providing an integrated solution capable of screening data with insufficient quality as well as further testing of the algorithm on increasingly noisy data.

In conclusion, the developed ANN yielded a high performance for the detection and localization of vessel-occlusions on CT-angiography in patients with AIS and substantially outperformed two currently available CE- and FDA-approved commercial software solutions during pseudo-prospective external benchmarking in consecutive patients with suspected AIS from four different hospitals during a 6-month timeframe. We provide an imaging platform (https://stroke.ccibonn.ai/) for online processing of medical imaging data with the developed ANN, including provisions for data crowdsourcing, which may serve as a basis for continuous refinements and as a blueprint to build robust and generalizable AI algorithms.

## Methods

### Study design and participants

The study of the imaging data was approved by the local ethics committee of the University of Heidelberg and the requirement to obtain informed consent was waived due to the due to the retrospective nature of the study and the thorough anonymization of the data.

In this retrospective multicentre study, we used CT-angiography data from 1179 patients previously treated at Heidelberg University Hospital (Heidelberg cohort) to develop and train a one-stage object detection ANN for detecting and localizing vessel occlusions on CT-angiography. The Heidelberg cohort included 800 consecutive patients with AIS and confirmed vessel occlusion on CT-angiography who subsequently underwent EVT between 03/2010 and 02/2020, as well as 379 consecutive patients with a suspected diagnosis of stroke but no vessel occlusion (control group) who underwent CT-angiography between 10/2019 and 02/2020. Pseudo-prospective external testing of the ANN was performed onto two different datasets, and namely (i) the FAST cohort, with 358 consecutive patients who underwent CT-angiography between 01/2022 and 06/2022 for suspected AIS at three primary/secondary care hospitals of the regional stroke consortium Rhine-Neckar with acute teleneurology/teleradiology coverage through the Heidelberg University Hospital, and the UKB cohort, with 323 patients who underwent CT-angiography between 09/2020 and 04/2021 for suspected AIS at the Department of Neuroradiology of the Bonn University Hospital.

Figure 1 depicts the flowchart with the inclusion and exclusion criteria for patients in the Heidelberg, FAST and UKB cohorts. All patients underwent multimodal CT, including CT-angiography. The scanner and acquisition parameters are shown in Supplementary Table 3.

### Procedures

Figure 1 depicts the flowchart of the procedures performed for training, model development and testing of the ANN. For the Heidelberg cohort, imaging data was exported from the PACS system and converted to the NifTI file format using dcm2niix (https://github.com/rordenlab/dcm2niix). All data was visually inspected, and cases presenting insufficient imaging quality were excluded (e.g., movement artifacts, insufficient vessel opacification, etc.). Vessel occlusions were then labeled by ES and reviewed by GB, a neuroradiology resident with 6 years of experience, and PV, a board-certified neuroradiologist with 10 years of experience, using ITK-SNAP (http://www.itksnap.org/). All vessel occlusions within the CT-angiography acquisition were labeled using a spherical 3D-ROI with 15 (MeVOs) or 30 (LVOs) voxels of diameter, placed with its center at the most proximal point of loss of contrast on one axial slice; the segmentation was then automatically propagated from the centerpoint in the 3D-plane. Both the treated occlusion and incidental findings were included in the labeling. The original radiological report was reviewed in all cases to improve robustness. Four main classes of occlusions were defined:

A. Anterior LVOs—occlusions in the common carotid artery (CCA), internal carotid artery (ICA), M1-segment of the middle cerebral artery (MCA) and A1-segment of the anterior cerebral artery (ACA)^19^
B. Anterior MeVOs—occlusions of the M2-/M3-segment of the MCA, A2-/A3-segment of the MCA^20^
C. Posterior LVOs—occlusions in the vertebral artery (VA), basilar artery (BA) and the P1-segment of the posterior cerebral artery (PCA)^19^
D. Posterior MeVOs—occlusions of the P2/3-segment of the PCA^20^

Next, patients with vessel occlusion were randomly split on a per-class basis into a training set (75%) and test set (25%) to maintain the distribution of occlusion locations. Within the test set, a 1:1 distribution between patients with vs. without vessel occlusion was established i.e., the same number of patients without vessel occlusion was added to the test set, whereas the remaining patients without vessel occlusion were added to the training set.

Detecting objects based on coarse annotations is a fundamental problem of computer vision and can be tackled with various methods. Here, we based our study on the use of RetinaNet^21,22^, a single-stage object detector that is both simple in design and provides a solid foundation for robust performance across various clinical problems^23^. A confidence threshold for the ANN prediction was determined on the training set by maximizing the F2-score, thereby putting more attention on minimizing false-negatives rather than false-positives. Performance was evaluated using 5-fold cross-validation on the training set and using the ensemble model for predicting the test set.

For the FAST cohort, imaging data from three regional hospitals (located in Mosbach, Sinsheim and Heppenheim - Germany) were previously sent to Heidelberg University Hospital for teleradiological reporting and therefore available in our local PACS system. Imaging data was exported in batch from the PACS system using ADIT (https://github.com/radexperts/adit) and converted to the NifTI file format using dcm2niix. Patients presenting insufficient data quality or missing data were excluded. To simulate a realistic usage of the developed ANN within a clinical scenario, CT-angiography data were then processed through the ANN (previously developed onto the Heidelberg cohort) to detect vessel occlusions, producing both labels and confidence scores (Fig. 2).

#### Commercial software comparison

Benchmarking of the developed ANN was performed against two FDA-cleared and CE-marked commercial software solutions which are currently available for purchase on the market (Software #1 and Software #2, respectively). Both software were tested on the UKB cohort, but only Software #2 could be tested onto the FAST dataset due to contractual limitations. The software names are blinded throughout the paper and cannot be disclosed at any given point; information on the architecture or mechanisms used by the software to detect the vessel occlusions are also not available due to the proprietary nature of the software solutions. Briefly, the software provided binary predictions and localizations of vessel occlusions, without further measures of uncertainty or confidence scores. Comparisons were performed visually by GB and reviewed by PV and UN; disagreements were resolved through consensus discussion. Written reports within the PACS system were referenced during the review process to increase accuracy and avoid misdiagnosis.

In order to provide a fair comparison with commercial software, which by design were both limited to detecting occlusions in the anterior circulation only, we analyzed all patients by considering only the detection of occlusions in the anterior circulation, and specifically limiting the analysis to occlusions in the internal carotid artery (ICA) and the M1-segment of the middle cerebral artery for LVOs, and in the M2- and M2-segment of the middle cerebral artery for MeVOs. Findings were considered correct as long as labeled on the correct vessel, without considering the precise localization of the occlusions, in order to provide a fair comparison between the software. Findings labeled in vascular territories not considered by the commercial software (e.g., posterior circulation, anterior cerebral artery) were ignored. McNemar’s test was used to compare specificity and sensitivity; comparison of relative predictive values was used instead to compare PPV and NPV (rpv.test function of R’s DTComPair package).

### Statistical analysis

In order to provide a full analysis of the capabilities of our algorithm while increasing comparability to previous studies^5–10,14,16,24–34^, the evaluation of the models was divided into (i) object-level and (ii) patient-level evaluation.

We performed automated and quantitative evaluation by using the expert-generated segmentation masks and the predicted bounding boxes from the CNN as input. Specifically, as further explained, the localization of a vessel occlusion was referred to as correct if the Intersection over Union (IoU) between the predicted bounding box exceeded 0.10^27,28^.

Briefly, for the calculation of patient-level performance the localization information was ignored, and patient-level classification results were produced by selecting the maximum of the predicted confidence scores. All CTA scans with at least one marked VO were then considered positive findings. The AUC was then used to evaluate the continuous predictions while sensitivity, specificity, PPV, and NPV were calculated at the same confidence thresholds as the object level evaluation. Only patients with a single VO were included in the patient-level evaluation when performance was assessed for each subgroup separately. Bootstrapping with 1000 iterations was used to determine the bootstrap percentile with 95% confidence intervals for the free-response operating characteristics (FROC^35^) and all other performance estimates.

FROC^36^ are a commonly found metric to evaluate CAD systems, and assesses the sensitivity at multiple working points and with a varying number of false-positive predictions per image. To obtain a single performance score from the entire curve, the sensitivity values at [1/8, 1/4, 1/2, 1, 2, 4, 8] were averaged. These values were selected in accordance with previous publications of CAD tools^35,37^ and account for the need for methods with high sensitivity in a screening setting while rewarding a low number of false positives. To account for the cubic decline of the IoU in three dimensional data and the coarse annotation of the RoI, the localization of a VO was referred to as correct if the IoU between the predicted bounding box and ground truth bounding box exceeded 0.10^23,38^.

For the object-level evaluation, localization information was maintained, and the localization of a vessel occlusion was referred to as correct if the IoU between the predicted bounding box and ground truth bounding box exceeded 0.10^39^. Duplicate predictions of the same VO were considered false positives. Since the detection task was formulated as a binary detection task (differentiating vessel occlusions from background), false-positive predictions could not be assigned to a respective subgroup. Subgroup analysis on the object-level was thus performed by computing sensitivity for each subgroup separately while the number of false positives were counted across all subgroups.

As further listed in the results, it also became apparent through the visual case review process that the network was also focusing on high-grade stenosis (HGS), and labeling these as false positive occlusions. High-grade stenoses constitute a clinically relevant vessel pathology which can cause stroke symptoms at presentation and require additional considerations when evaluating stroke CTAs, as well as when planning the following intervention. Therefore, we conducted a separate sub-analysis by also documenting high-grade vessel stenoses. HGS were labeled with the same procedures as VOs, and were considered high-grade if above 70% of the vessel lumen; measurements at the carotid bifurcation were performed according to the NASCET trial standard^40^. Within this sub-group analysis, previous false positives labels on confirmed high-grade stenoses were considered true positives. Conversely, any missed high-grade stenosis was considered a false negative both at case- and object-level. Cross-referencing with the radiological report was always performed during the labeling procedures to increase accuracy.

### Network training: image pre-processing

The target spacing was set to the median spacing of the training cohort (namely, 0.5 mm × 0.453 mm × 0.453 mm). Since the single voxel density values of CT scans are implicitly measured on an absolute scale expressed in Hounsfield units, we employed a global normalization scheme for all cases in order to avoid loss of information^41^; specifically, the statistical properties of the voxel intensities such as mean, standard deviation, and percentiles were collected across the entire training dataset and were used to clip the voxel intensities to their 0.5 and 99.5 percentiles followed by z-score normalization^41^.

### ANN architecture

The RetinaNet^21^ architecture consists of three main components: the encoder network which consecutively downsamples the image to extract features on multiple resolutions, the decoder network which progressively upsamples the obtained features to combine coarse (low resolution) with fine grained (high resolution) features and the detection heads which are responsible for classifying and regressing the anchors. The ANN receives three-dimensional input patches with [192, 128, 128] voxels for processing. A detailed overview of the architecture can be found in Supplementary Fig. 1.

The encoder network utilizes 3 × 3 × 3 convolutions, Instance Normalisation and Leaky Rectified Linear Units to extract features. Strided convolutions at the beginning of each resolution stage are used to downsample the feature maps. The four deepest (i.e., lowest resolution) feature maps are used for further processing by the decoder network.

A Feature Pyramid Network^42^ is used to recombine information from different resolutions. First, each feature map is processed by a 1 × 1 × 1 convolution to reduce the number of channels to 128. Transposed convolutions are used to progressively upsample them and element wise addition is used to combine the features.

These feature maps are fed to a set of shared convolutions, usually referred to as the detection head. It is responsible to classify and regress the predefined set of anchors and consists of 3 × 3 × 3 convolutions, Group Normalisation^43^, and Leaky Rectified Linear Units.

### Network training details

Anchors are an essential concept of several commonly used object detectors, as they act as initial estimates of objects and are used to formulate the detection task as a classification and regression problem. To account for the differently sized annotations, two anchors of size [8, 10, 10] and [15, 14, 14] were used during our experiments, which were derived by the planning procedure of nnDetection^23^. The assignment of ground truth objects to anchors during the training was conducted via Adaptive Training Sample Selection^44^. Binary cross-entropy loss was used to train the classification branch of the detection head and the regression branch was trained with the smooth L1 loss^45^.

To reduce overfitting and artificially increase the diversity of the training samples, online data augmentation was utilized throughout the entire training. In order to avoid artifacts at the edges when applying spatial augmentations, a patch size of [328, 249, 295] was extracted from the CTA scan and cropped to the training patch size of [192, 128, 128] after the spatial augmentations were applied. We utilized the same set of augmentations as nnU-Net^41^ except dropping the Simulation of Low-Resolution Samples due to the observation of a slightly reduced performance when using it.

The ANN was trained with Focal loss^21^ and smooth L1 loss^45^ in a fivefold cross-validation fashion to differentiate between background and labeled VOs. The 5 folds were generated by generating stratified randomized folds considering all available classes, and the overall least frequent class present in a patient was used as a basis for randomization. During our cross-validation experiments, we found that training for 60 epochs with 2500 batches each while using SGD with Nesterov Momentum^23,41^ to update the weights achieved the highest FROC score on our dataset. Here, the last 10 epochs were used for Stochastic Weight Averaging to further optimize the final model^46^. Training was performed on patches to overcome the memory limitations caused by the 3D model configuration; patches were set to a size of 192 × 128 × 128 voxels, with a batch size of 8, and were sampled from the CTA scans while ensuring an equal number of foreground and background patches per batch.

Since occlusion annotation was depending on the occurrence of intravascular loss of contrast, multiple annotated occlusions may be present in a single patient. Frequently this was caused by tandem occlusions, especially simultaneous occlusion of the internal carotid artery and the middle cerebral artery^47^. Nevertheless, coincidental findings as well as combinations of recent and preexisting vessel occlusions were also included.

#### Network training: selection of the confidence threshold

To provide an analysis at one specific working point, a cutoff had to be determined for the continuous confidence scores produced by the network. At the time of testing, one model from each fold was then ensembled to form a single prediction. Since the models need to agree on the prediction, the distribution of the confidence scores between the fivefold cross-validation and the test set are implicitly different. To account for this shift, we designed an additional experiment on the training set data.

The training cohort (*n* = 835) was separated into a mini-training (*n* = 418) and mini-evaluation (*n* = 417) cohort. The mini-evaluation dataset contained 207 control patients and 210 patients with at least one vessel occlusion, see Supplementary Fig. 2. This experiment used the same hyperparameters for training and class balancing procedure to generate the folds as the primary experiment. After predicting the mini-evaluation dataset, the cutoff was determined to maximize the F2 score on the object level. The F2 score was previously used by ref. ^48^ to assess object level performance and was chosen in our experiment to adjust the tradeoff between sensitivity and precision. The best F2 score of 0.74 on the mini-evaluation set was reached at a confidence cutoff of 0.647, see Supplementary Fig. 3.

### Prediction details

During testing and fivefold cross-validation, each patient was predicted via a sliding window scheme with 50% patch overlap. To suppress duplicate predictions of the same vessel occlusion, Non-Maximum Suppression with an Intersection Over Union Threshold of 0.3 was applied and predictions that were close to the center of the patch received a higher weighting than predictions close to the border. For each model, all predictions with a confidence score above 0.2 were selected for further ensembling. Weighted Box Clustering (WBC)^38^ (without restrictions on the area of the predictions) was used to combine predictions from the different models. Predictions that exceeded an Intersection over Union of 0.4 were considered clusters and merged into a single prediction. All bounding boxes which had any axis smaller than 7 voxels were removed from the final set of predictions.

Model inference was performed on a NVIDIA DGX A100 (NVIDIA, Santa Clara, CA, USA) by using four GPUs with a sliding window scheme and 50% patch overlap^23^. Non-maximum suppression was applied in order to remove duplicate predictions from neighboring patches, with predictions near the center of a patch weighted with higher importance than predictions close to the borders. The final five models (one from each fold) were ensembled via WBC^38^. Postprocessing parameters were determined by empirical hyperparameter tuning on the training set as previously described in the literature^23^.

### CTA phase correlation

To assess possible correlations between CTA scan phase and detection performance, as reported before^8^, test set scans were classified into five different groups, ranging from arterial (early arterial, peak arterial) and equilibrium to venous phase (peak venous, late venous). Classification was performed following a previously published method^49^.

#### Hardware

The prediction of the test cases was performed on a DGX A100 with Ubuntu 20.04.4 LTS. The computer was equipped with two AMD EPYC 7742 with 64 physical cores each as its CPU and 1-TB of Random Access Memory (RAM). Four NVIDIA A100 with 40-GB of Video Random Access Memory (VRAM) were used as the GPUs. The preprocessing step to normalize the data is the predominant bottleneck when multiple GPUs are used to predict the ANNs.

Distributed Data Parallel from PyTorch was used to predict a subset of the extracted patches for the sliding window approach on each GPU. The predictions from each GPU were gathered before the ensembling step. This approach allows for a flexible number of GPUs to be used to predict each patient with little synchronization overhead between different processes.

### Reporting summary

Further information on research design is available in the Nature Portfolio Reporting Summary linked to this article.

## Supplementary information

Supplementary Information File

Reporting Summary

## Source data

Source Data

## Data Availability

The imaging data used for the study are protected and are not available due to data privacy laws. Access can be requested by sending a request to the corresponding author for academic purposes. The corresponding author will process requests within 3 months and follow-up to the requesting party. Any request will be pending prior approval and revision by the Heidelberg University Hospital as owner of the data and the Ethics Committee of the Medical Faculty of the University of Heidelberg, which retain all rights to deny access. The de-identified data generated during and/or analyzed during the current study are provided as source data file; any further de-identified data are available from the corresponding author on request. The names of the two commercial software cannot be disclosed at any given point. Source data are provided with this paper.

## Code availability

The development of the ANN is based on our previously published and publicly available nnDetection method available via https://github.com/MIC-DKFZ/nnDetection. All other codes can be requested from the corresponding author, which will process requests within 3 months and follow-up to the requesting party. The trained model to generate the results is deployed on our publicly available website https://stroke.ccibonn.ai/ and allows online processing of CT-Angiography data with the developed ANN.
