# Supplementary material for: Deep-learning based detection of vessel occlusions on CT-angiography in patients with suspected acute ischemic stroke
Source: Nat Commun. 2023 Aug 15;14:4938. doi: 10.1038/s41467-023-40564-8 (PMC10427649; doi:10.1038/s41467-023-40564-8)
Supplement: Supplementary file 1 — Supplementary Information File [file 41467_2023_40564_MOESM1_ESM.pdf]

Supplementary Information File

Supplementary Notes: Software Versions

- Numpy:: 1.21.4
- Batchgenerators:: 0·21
- Scipy: 1.73
- Python: 3.8: python-3.8.12
- PyTorch: 1.10: 1.10·0+cu113
- Pytorch Native Mixed Precision
- CUDA: CUDA Version: 11.4
- nnU-Net: 1.6.6
- nnDetection: 0·1
- dcm2niiX version: 1.0·20200427
- ITK SNAP: 3.8.0

Supplementary Figures

Supplementary Figure 1. The detailed network architecture of the utilized RetinaNet. The numbers next to each convolution describe the output size of the features (channel first format). The ANN can be divided into three main components: the encoder on the left side, the decoder in the middle, and a detection head which is shared across multiple resolution levels. Abbreviations: conv: convolution, IN: Instance Normalisation, GN: Group Normalisation, LReLU: Leaky Rectified Linear Unit.

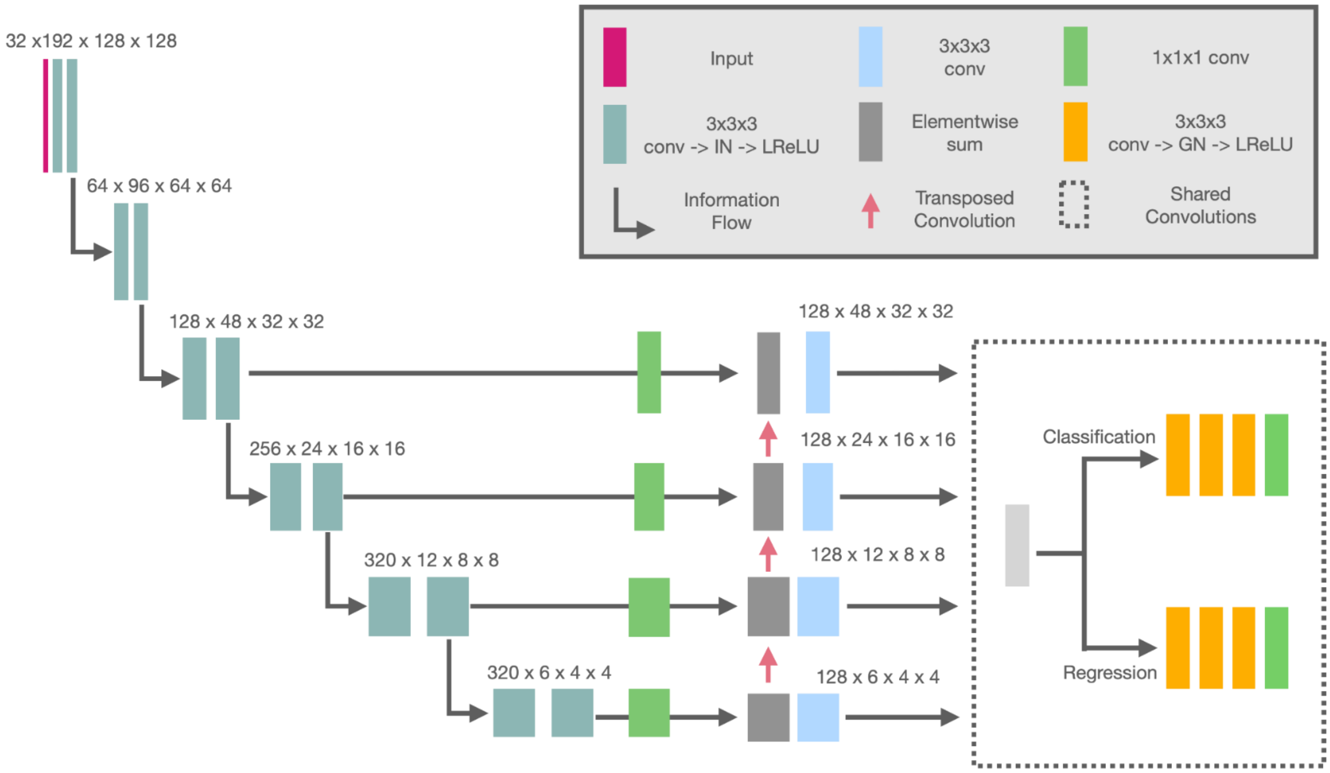

Supplementary Figure 2. Flowchart of the experimental setup to determine the confidence cutoff by using a mini-training and mini-evaluation set.

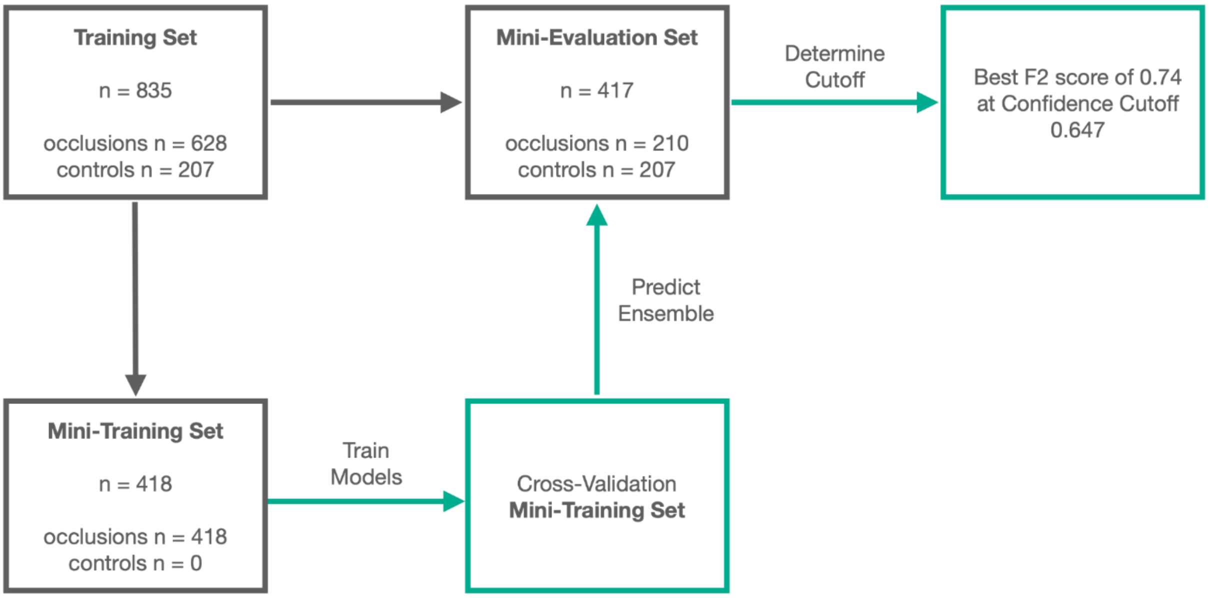

Supplementary Figure 3. Visualization of the object level F2 score on the mini-evaluation set. The best score of 0·74 was reached at a confidence cutoff of 0·647. Source data are provided as a Source Data file.

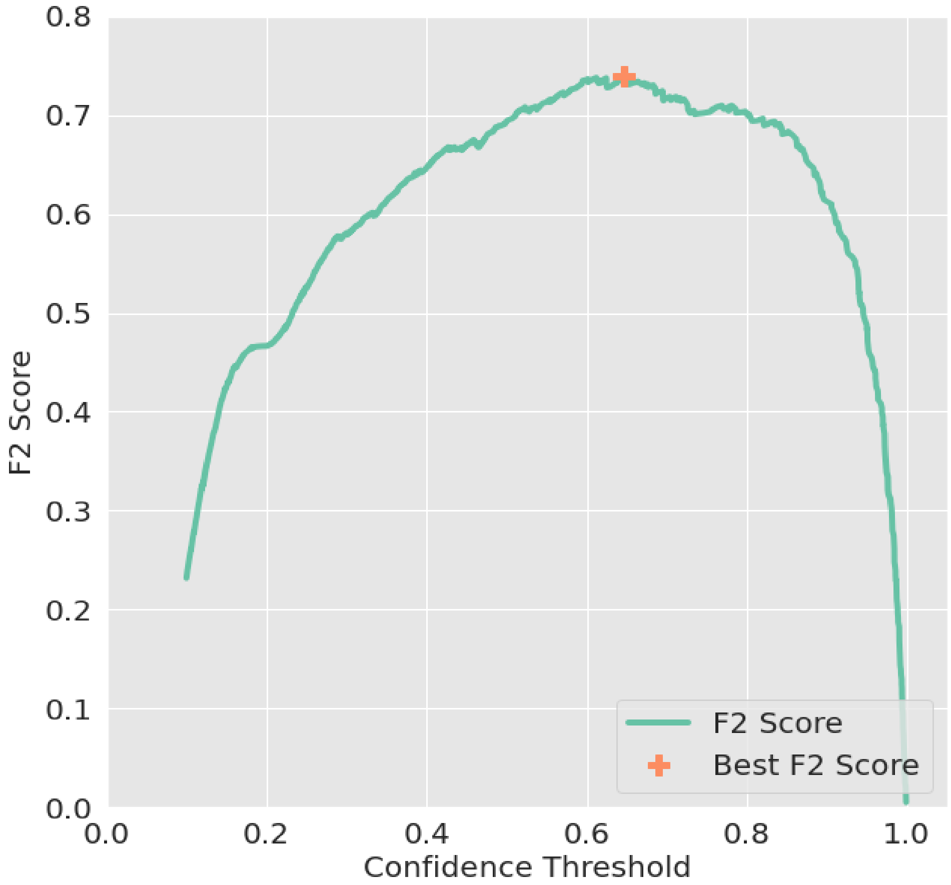

Supplementary Figure 4. Box plot to visualize the processing time for each patient in the test set of the Heidelberg cohort (n=344). The centre line depicts the median valuewhile the box extends from the first quartile to the third quartile and the whiskers further extend by 1.5 times the inter-quartile range. Any points exceeding these limits are drawn as dots. Source data are provided as a source data file.

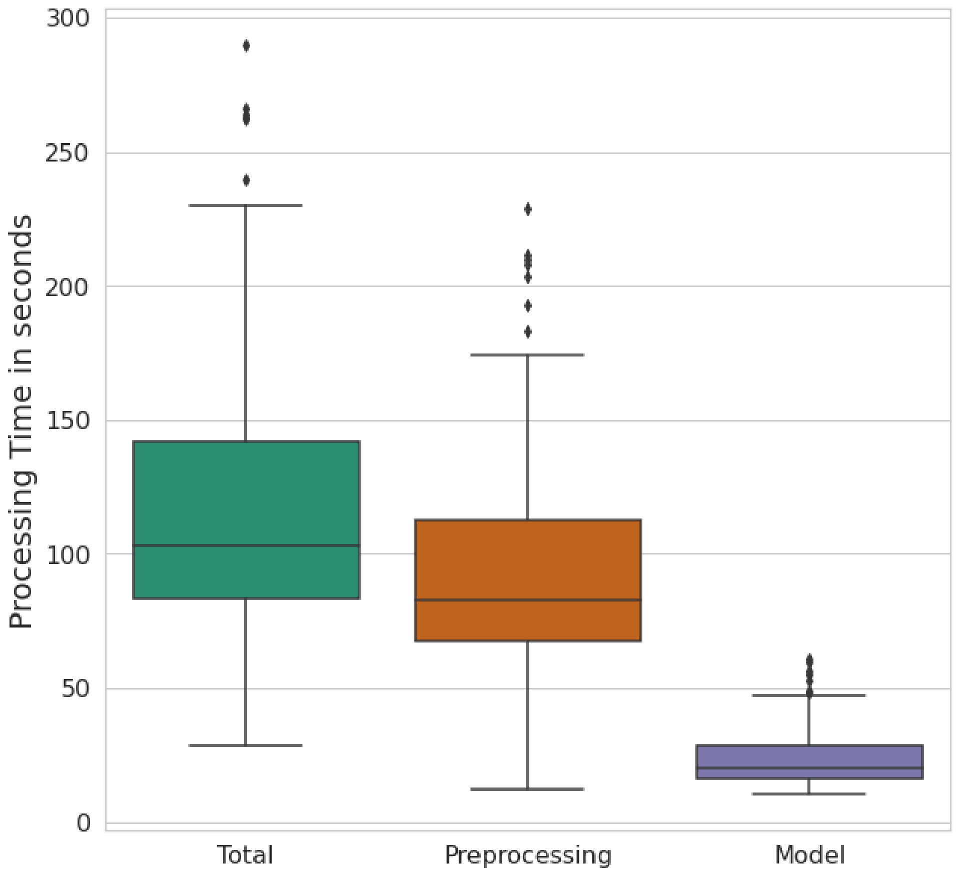

Supplementary Figure 5. Free-response operating characteristic (FROC) for each VO subgroup on the internal test set; Abbreviations: VO - vessel occlusion, LVO - large vessel occlusion, MeVO - medium vessel occlusion. Source data are provided as a source data file.

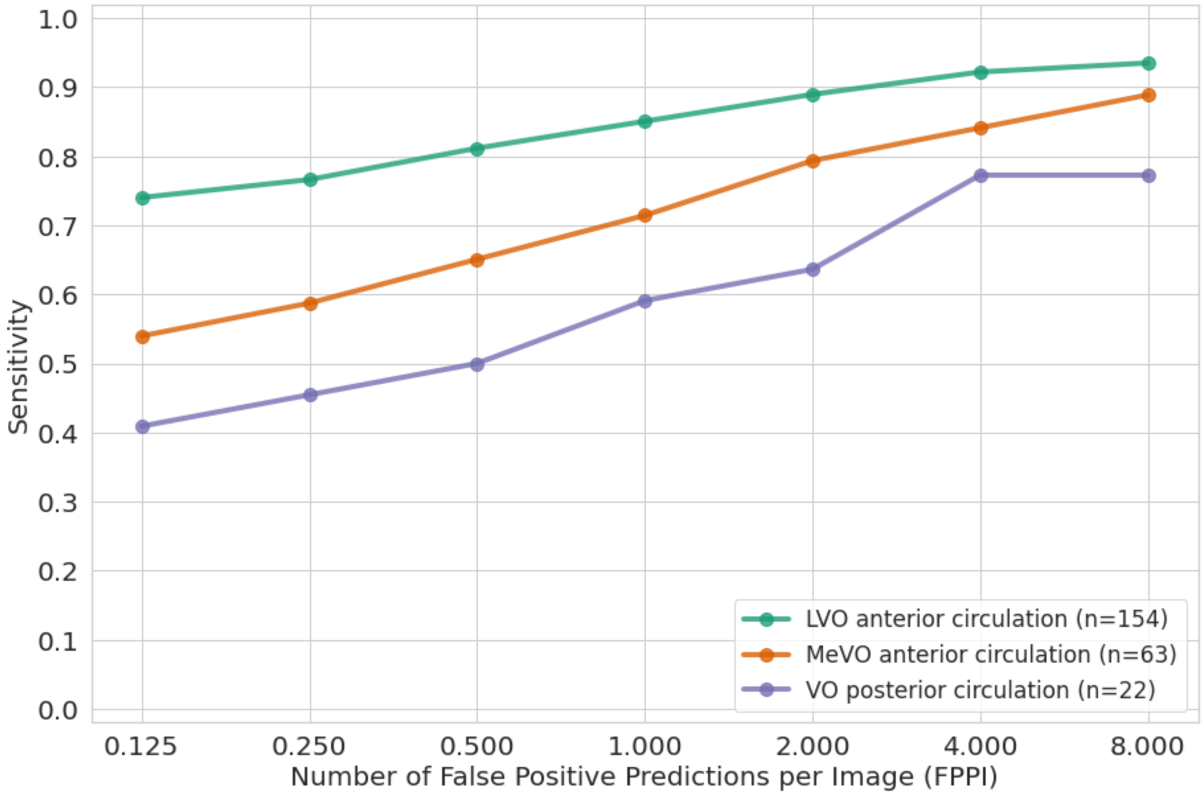

Supplementary Figure 6. Free-response operating characteristic for each VO subtype on the test set of the Heidelberg cohort. Source data are provided as a source data file.

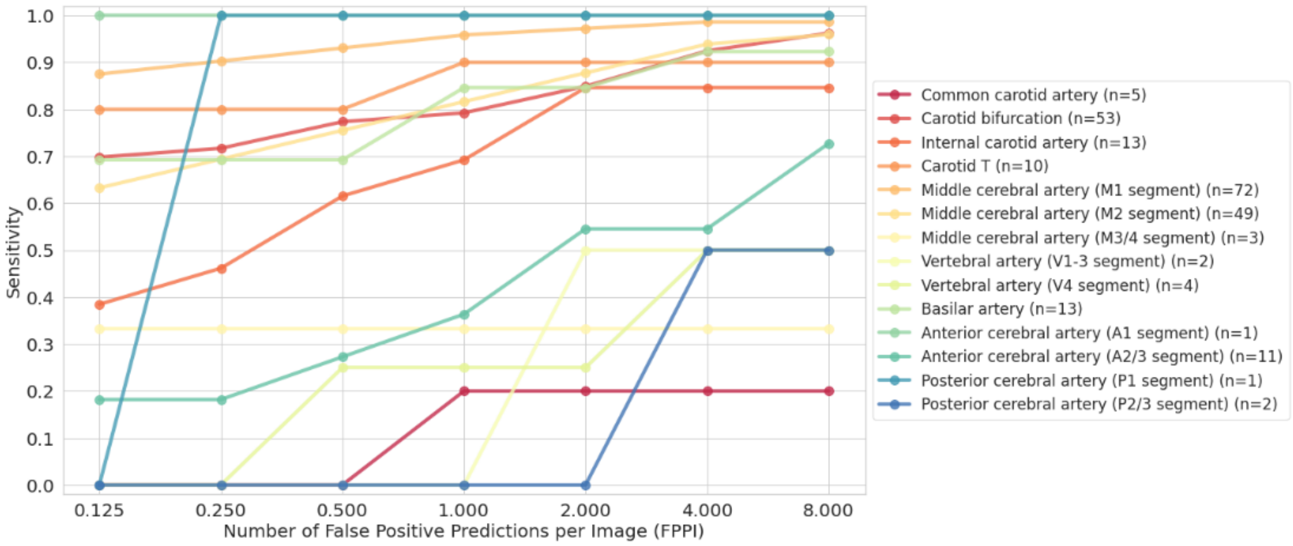

Supplementary Figure 7. Correlation of number of cases per occlusion site in the training dataset of the Heidelberg cohort vs. the average confidence score predicted by the ANN in the test dataset of the Heidelberg cohort. 95% confidence intervals are computed via 1000 iterations of bootstrapping and drawn as the light grey area. A two-sided Wald Test with t-distribution is used to test for statistical significance. Source data are provided as a source data file.

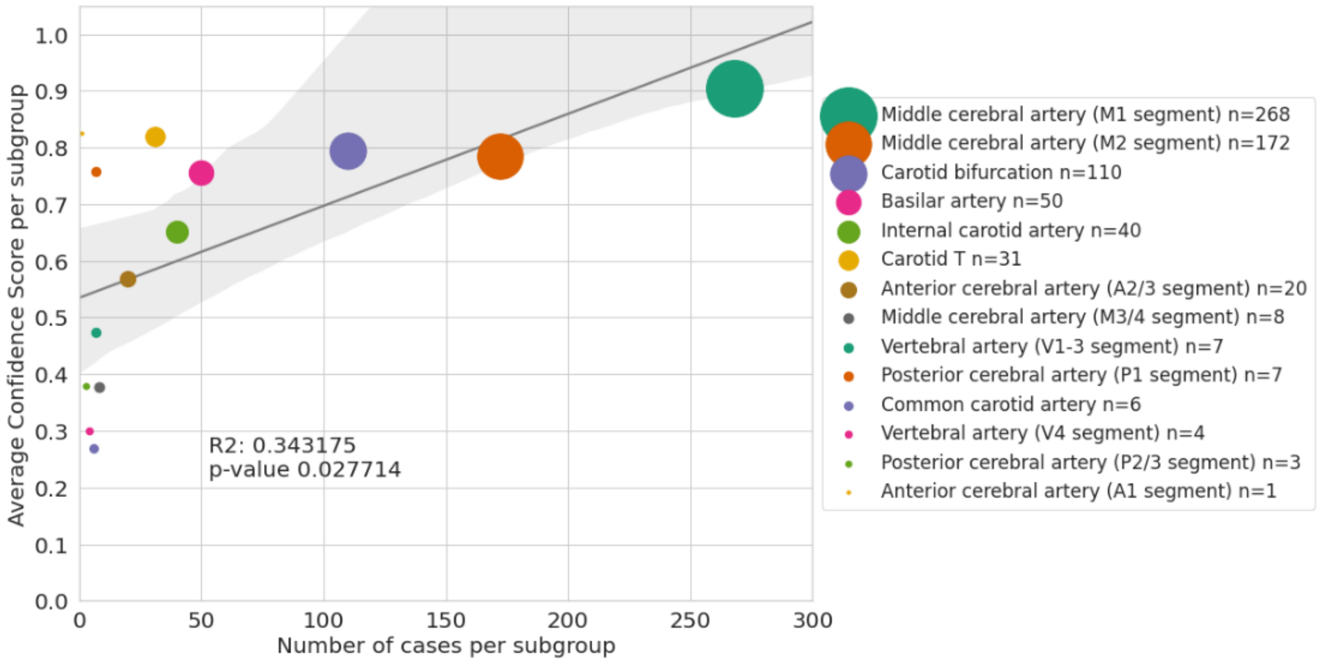

Supplementary Figure 8. Free-response operating characteristic for each VO subgroup in the 5-fold cross-validation (training set) of the Heidelberg cohort; Abbreviations: VO - vessel occlusion, LVO - large vessel occlusion, MeVO - medium vessel occlusion. Source data are provided as a source data file.

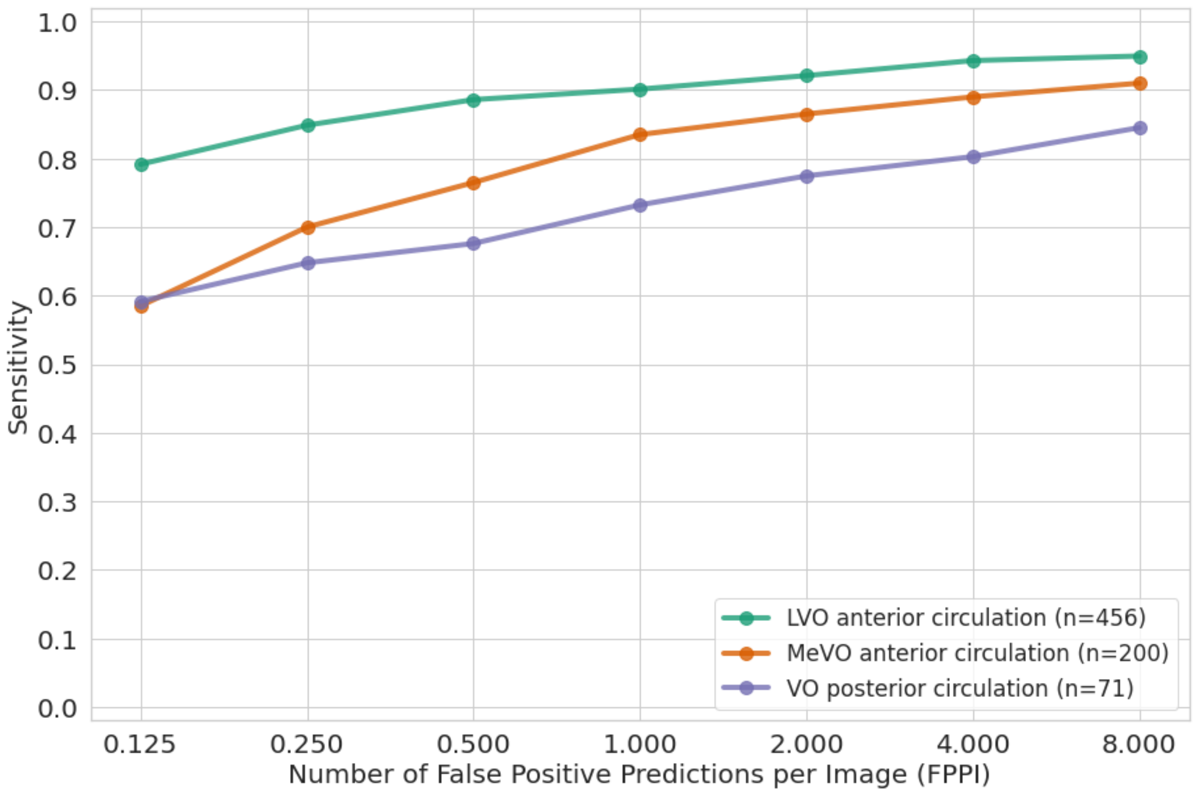

Supplementary Tables

Supplementary Table 1. Occlusion types per study group (LVO: large vessel occlusion, MeVO: medium vessel occlusion (*Pearson's chi-squared test was used to compare the distribution of individual occlusion types between training and test set). P-values considered significant are highlighted in bold.

	**Heidelberg cohort (n=966)**			**FAST cohort (n=327)**	**UKB Cohort (n=323)**	**p-value (FAST vs. UKB)**
	**Training set (n=727/966; 75%)**	**Test set (n=239/966; 25%)**				
Occlusion positive	628/727	172/239		52/327 (16%)	85/323 (26%)	**0·002**
**Anterior circulation**	656 (90%)	217 (91%)		45 (14%)	68 (21%)	**0·019**
**LVO**	456 (63%)	154 (64%)		30 (9%)	43 (13%)	0·122
Common carotid artery (CCA)	6 (1%)	5 (2%)		2 (1%)	0 (0%)	1
Carotid bifurcation	110 (15%)	53 (22%)		11 (3%)	15 (5%)	0·527
Internal carotid artery (ICA)	40 (6%)	13 (5%)		2 (1%)	7 (2%)	0·173
Carotid-T	31 (4%)	10 (4%)		0 (0%)	2 (1%)	0·473
Anterior cerebral artery (ACA) - A1 segment	1 (0%)	1 (0%)		0 (0%)	0 (0%)	-
Middle cerebral artery (MCA) - M1 segment	268 (37%)	72 (30%)		16 (5%)	19 (6%)	0·700
**MeVO**	200 (28%)	63 (26%)		15 (5%)	29 (9%)	**0·038**
Anterior cerebral artery (ACA) - A2/3 segment	20 (3%)	11 (5%)		2 (1%)	2 (1%)	1

Middle cerebral artery (MCA) - M2 segment	172 (24%)	49 (21%)		9 (3%)	22 (7%)	**0·025**
Middle cerebral artery (MCA) - M3/4 segment	8 (1%)	3 (1%)		4 (1%)	5 (2%)	0·985
**Posterior circulation**	71 (10%)	22 (9%)		13 (4%)	19 (6%)	0·346
**LVO**	68 (9%)	20 (8%)		9 (3%)	15 (5%)	0·284
Basilar artery	50 (7%)	13 (5%)		2 (1%)	7 (2%)	0·173
Posterior cerebral artery (PCA) - P1 segment	7 (1%)	1 (0%)		2 (1%)	4 (1%)	0·671
Vertebral artery (VA) - V1-3 segment	7 (1%)	2 (1%)		2 (1%)	3 (1%)	0·989
Vertebral artery (VA) – V4 segment	4 (1%)	4 (2%)		3 (1%)	1 (0·3%)	0·625
**MeVO**	3 (0%)	2 (1%)		5 (2%)	4 (1%)	1
Posterior cerebral artery (PCA) - P2/3 segment	3 (0%)	2 (1%)		4 (1%)	4 (1%)	1

Supplementary Table 2. Patient demographics in the Heidelberg, FAST and UKB cohort. IQR: interquartile range (Pearson's chi-squared test was used for comparing the distribution of categorical variables and Kruksal Wallis test were used for comparing continuous variables between the training and test set)

	**Heidelberg cohort**		**FAST cohort**	**UKB Cohort**	**p-value**
	**Training dataset**	**Test dataset**			
**Occlusion positive**	628	172	52	85	
**Sex**					
Male (%)	305 (49%)	89 (52%)	26 (49%)	46 (54%)	0·735
Female (%)	323 (51%)	83 (48%)	27 (50%)	39 (46%)	
**Age**					
Median (IQR)	78.0 (15.5)	75.5 (18.0)	82 (15.5)	76.9 (17.06)	**0·019**

Supplementary Table 3. Imaging features. B10f, B20f, B25f, B30f, B31f, Bv40f, B46f: filtered back projection algorithm with very smooth (B10f) to sharp (B46f) body kernel; I26f, I30f: iterative reconstruction algorithm with a medium smooth body kernel; D26f: medium smooth dual-energy convolution kernel; H30f: medium smooth head convolution kernel; CTA: computed tomography angiography; kVp: kilovoltage peak. Contrast bolus and flow rate information was available for only n=74 patients within the FAST cohort. Contrast bolus information was not available in the DICOM headers for the UKB cohort; CTA here was acquired with an automated bolus injection of 50ml Iohexol (Accupaque 350, GE Healthcare) and 350ml NaCl-Solution at a Flow-Rate of 4ml/s.

	Heidelberg cohort (n=1179)	FAST Cohort (n=327)	UKB Cohort (n=323)
**Scanner Model**			
Siemens SOMATOM Definition AS	1136 (96%)	124 (38%)	0 (0%)
Siemens SOMATOM X.cite	0 (0%)	36 (11%)	0 (0%)
Siemens Sensation 16	31 (3%)	0 (0%)	0 (0%)
Siemens Sensation 40	0 (0%)	167 (52%)	0 (0%)
Siemens Sensation Open	7 (1%)	0 (0%)	0 (0%)
Siemens SOMATOM Definition Flash	4 (0%)	0 (0%)	0 (0%)
Siemens SOMATOM Definition	1 (0%)	0 (0%)	0 (0%)
Philips IQon – Spectral CT	0 (0%)	0 (0%)	323 (100%)
**Convolution Kernel**			
B26f	746 (63%)	0 (0%)	0 (0%)
I30f	382 (32%)	0 (0%)	0 (0%)
H30f	31 (3%)	85 (26%)	0 (0%)
Hv40f	0 (0%)	1 (3%)	0 (0%)
B25f	0 (0%)	42 (13%)	0 (0%)
B30f	7 (1%)	0 (0%)	0 (0%)
B31f	0 (0%)	40 (12%)	0 (0%)
Bv40f	0 (0%)	35 (11%)	0 (0%)
D26f	4 (0%)	0 (0%)	0 (0%)
I26f	4 (0%)	124 (38%)	0 (0%)
B46f	3 (0%)	0 (0%)	0 (0%)
B10f	1 (0%)	0 (0%)	0 (0%)

B20f	1 (0%)	0 (0%)	0 (0%)
B	(0%)	(0%)	323 (100%)
**Slice Thickness**			
0·75 mm	1110 (94%)	4 (1%)	0 (0%)
0·80 mm	0 (0%)	40 (12%)	0 (0%)
0·6 mm	60 (5%)	0 (0%)	0 (0%)
1.0 mm	8 (1%)	209 (64%)	323 (100%)
1.2 mm	0 (0%)	38 (12%)	0 (0%)
1.5 mm	0 (0%)	36 (11%)	0 (0%)
2.0 mm	1 (0%)	0 (0%)	0 (0%)
Slice Thickness, median [IQR]	0,75 [0.75-0.75]	1.00 [1.00-1.00]	1.00 [1.00-1.00]
**kVp** median (min-max)	120 (80-140)	171 (133-480)	194 (155-244)
**Exposure** [mAs] median (min-max)	152 (49-300)	61 (47-200)	152 (121-191)
**Contrast bolus** [ml] median (min-max)	65 (45-66)	80 (40-80)	Not Available
**Contrast flow rate** [ml/s] median (min-max)	4 (3-4)	4 (4-4)	Not Available

Supplementary Table 4. Distribution of patients with multiple occlusions and tandem occlusions. Only unilateral occlusions have been outlined here. “Others” may contain patients with bilateral occlusions. Percentages refer to the number of patients with occlusions in the training and test set, respectively.MCA: middle cerebral artery. Pearson's chi-squared test was used for comparing the distribution of occlusions. No relevant differences were observed in the distribution of Carotid Bifurcation + MCA-M1 (p=0.230), Carotid Bifurcation + MCA-M2 (p=0.396), Multiple MCA-M2 (p=0.770). The dataset differed instead in the distribution of different (other) cases with multiple occlusions (p=0.008), which was also reflected when analyzing all possible multiple occlusions (p<0.001). P-values considered significant are highlighted in bold.

	**Training set**	**Test set**	**FAST**	**UKB**	**p**
**Patients with multiple occlusions / tandem occlusions**	96 (15%)	56 (33%)	4/53 (7%)	9/85 (11%)	**<0·001**
Carotid bifurcation + MCA-M1	30 (5%)	15 (9%)	2 (4%)	1 (1%)	0·230
Carotid bifurcation + MCA-M2	12 (2%)	8 (5%)	1 (2%)	1 (1%)	0·396
Multiple MCA-M2	4 (1%)	2 (1%)	0 (0%)	0 (0%)	0·770
Others	50 (8%)	31 (18%)	1 (2%)	7 (8%)	**0·008**

Supplementary Table 5. Comparison of the frequency distribution with regards to the computed tomography (CT) angiography acquisition phase between the HD cohort (test set) and the FAST cohort. Pearson's chi-squared test was used for comparing the distribution of contrast phase. The datasets presented significant differences in all the examined contrast phases (p<0.001), indicating the heterogeneity of the chosen data. P-values considered significant are highlighted in bold.

	**Acquisition phase of the CT angiography in the test sets**				
	**Early Arterial**	**Peak Arterial**	**Equili-brium**	**Peak Venous**	**Late Venous**
**HD cohort**	n = 258/344 (75%)	n = 39/344 (11%)	n = 40/344 (12%)	n = 4/344 (1%)	n = 3/344 (1%)
**FAST cohort**	n = 42/327 (13%)	n= 134/327 (41%)	n=114/327 (35%)	n=33/327 (11%)	n=4/327 (1%)
**UKB cohort**	n= 32/323 (10%)	n= 73/323 (23%)	n= 133/323 (41%)	n= 65/323 (20%)	n= 21/323 (7%)
**p-values**	**<0·001**	**<0·001**	**<0·001**	**<0·001**	**<0·001**

Supplementary Table 6. Patient-level performance for detection and localisation of vessel occlusions in the training set (5-fold cross-validation) of the Heidelberg cohort measured by AUROC, Sensitivity, Specificity, PPV and NPV; Abbreviations: VO - vessel occlusion, LVO - large vessel occlusion, MeVO - medium vessel occlusion, PPV - positive predictive value, NPV - negative predictive value

**Cohort**<br>**n = Num Patients**	**AUROC**	**Sensitivity**	**Specificity**	**PPV**	**NPV**
VO (n = 628) / Controls (n = 207)	0·96<br>[0·95, 0·97]	601/628<br>(96)<br>[94, 97]	159/207<br>(77)<br>[71, 82]	601/649<br>(93)<br>[91, 95]	159/186<br>(85)<br>[80, 90]
**Anterior Circulation**					
LVO (n=333) / Controls (n = 207)	0·98<br>[0·97, 0·99]	325/333<br>(98)<br>[96, 99]	-	325/373<br>(87)<br>[84, 90]	159/167<br>(95)<br>[92, 98]
MeVO (n = 149) / Controls (n = 207)	0·93<br>[0·90, 0·95]	136/149<br>(91)<br>[87, 95]	-	136/184<br>(74)<br>[67, 80]	159/172<br>(92)<br>[88, 96]
**Posterior Circulation**					
VO (n = 50) / Controls (n = 207)	0·93<br>[0·87, 0·97]	45/50<br>(90)<br>[82, 98]	-	45/93<br>(48)<br>[39, 58]	159/164<br>(97)<br>[94, 99]

Supplementary Table 7. Patient-level performance for identifying the presence of vessel occlusion in the test set of the Heidelberg cohort; Abbreviations: AUROC - area under the receiver operating characteristic, VO - vessel occlusion, LVO - large vessel occlusion, MeVO - medium vessel occlusion, PPV - positive predictive value, NPV - negative predictive value

**Cohort**<br>**n = Num Patients**	**AUROC**	**Sensitivity**	**Specificity**	**PPV**	**NPV**
VO (n = 172) /<br>Controls (n = 172)	0·96<br>[0·95, 0·98]	161/172<br>(94)<br>[90, 97]	142/172<br>(83)<br>[77, 88]	161/192<br>(84)<br>[79, 89]	142/153<br>(93)<br>[88, 96]
**Anterior Circulation**					
LVO (n=74) /<br>Controls (n = 172)	0·98<br>[0·97, 1.00]	73/74<br>(99)<br>[96, 100]	-	73/103<br>(71)<br>[61, 79]	142/143<br>(99)<br>[98, 100]
MeVO (n = 29) /<br>Controls (n = 172)	0·94<br>[0·89, 0·98]	25/29<br>(86)<br>[72, 97]	-	25/55<br>(45)<br>[33, 60]	142/146<br>(97)<br>[94, 100]
**Posterior Circulation**					
VO (n = 13) /<br>Controls (n = 172)	0·87<br>[0·73, 0·98]	10/13<br>(77)<br>[50, 100]	-	10/40<br>(25)<br>[11, 39]	142/145<br>(98)<br>[96, 100]

Supplementary Table 8. Object-level performance for detection and localisation of vessel occlusions in the training set (5-fold cross-validation) of the Heidelberg cohort; Abbreviations: VO=vessel occlusion; LVO: large vessel occlusion, MeVO: medium vessel occlusion; FROC=free-response operating characteristic; S=sensitivity; FFPI=Number of false positives per image; VO = vessel occlusion

**Cohort n=Num VO**	**FROC**	**S@0·5FPPI**	**S@1FPPI**	**S@Thresh**
VO (n = 727)	0·85 [0·82, 0·87]	0·83 [0·80, 0·86]	0·87 [0·84, 0·89]	0·85 [0·82, 0·88]
**Anterior Circulation**				
LVO (n = 456)	-	0·89 [0·85, 0·91]	0·90 [0·87, 0·93]	0·90 [0·87, 0·92]
MeVO (n = 200)	-	0·77 [0·70, 0·83]	0·84 [0·77, 0·89]	0·81 [0·74,0·86]
**Posterior Circulation**				
VO (n = 71)	-	0·68 [0·56, 0·78]	0·73 [0·63, 0·84]	0·70 [0·60, 0·80]

Supplementary Table 9. Object-level performance for detection and localisation of vessel occlusions in the test set of the Heidelberg cohort; Abbreviations: VO=vessel occlusion; LVO: large vessel occlusion, MeVO: medium vessel occlusion; FROC=free-response operating characteristic; S=sensitivity; FFPI=Number of false positives per image; VO = vessel occlusion

Cohort n=Num VO	FROC	S@0·5FPPI	S@1FPPI	S@Thresh
All (n = 239)	0·79 [0·73, 0·84]	0·74 [0·67, 0·81]	0·79 [0·73, 0·85]	0·73 [0·67, 0·79]
**Anterior Circulation**				
LVO (n = 154)	-	0·81 [0·74, 0·88]	0·85 [0·78, 0·91]	0·81 [0·74, 0·87]
MeVO (n = 63)	-	0·65 [0·53, 0·77]	0·71 [0·60, 0·82]	0·63 [0·51, 0·75]
**Posterior Circulation**				
VO (n = 22)	-	0·50 [0·31, 0·7]	0·59 [0·4, 0·78]	0·50 [0·31, 0·7]

Supplementary Table 10. Object-level performance for detection and localization of vessel occlusions in the FAST cohort. Abbreviations: VO=vessel occlusion; HGS= High-Grade Stenosis (>70%); LVO: large vessel occlusion, MeVO: medium vessel occlusion; PPV - positive predictive value

Metrics	FROC	S@0.5FPPI	S@1FPPI	S@Thresh
**Full dataset**				
VO (n = 58)	0.75 [0.65, 0.85]	0.76 [0.64, 0.86]	0.79 [0.68, 0.89]	0.72 [0.61, 0.83]
VO + HGS (n=96)	0.76 [0.68, 0.84]	0.75 [0.65, 0.84]	0.77 [0.67, 0.87]	0.73 [0.63, 0.82]
**Class: VO**				
**Anterior Circulation**				
LVO (n = 31)	-	0.84 [0.70, 0.96]	0.84 [0.70, 0.96]	0.77 [0.62, 0.91]
MeVO (n = 15)	-	0.87 [0.67, 1.00]	0.87 [0.67, 1.00]	0.87 [0.67, 1.00]
**Posterior Circulation**				
VO (n = 12)	-	0.42 [0.11, 0.71]	0.58 [0.25, 0.86]	0.42 [0.11, 0.71]
**Class: VO+HGS**				
**Anterior Circulation**				
LVO (n = 49)	-	0.80 [0.66, 0.92]	0.80 [0.66, 0.92]	0.76 [0.61, 0.88]
MeVO (n = 29)	-	0.86 [0.74 0.97]	0.86 [0.74 0.97]	0.86 [0.74, 0.97]
**Posterior Circulation**				
VO (n = 18)	-	0.44 [0.23, 0.70]	0.56 [0.31, 0.79]	0.44 [0.21, 0.69]

Supplementary Table 11. Object-level performance for detection and localization of vessel occlusions in the UKB cohort. Abbreviations: VO=vessel occlusion; HGS= High-Grade Stenosis (>70%); LVO: large vessel occlusion, MeVO: medium vessel occlusion; PPV - positive predictive value

**Cohort n=Num VO**	**FROC**	**S@0.5FPPI**	**S@1FPPI**	**S@Thresh**
VO (n = 89)	0.74 [0.66, 0.82]	0.73 [0.63, 0.82]	0.76 [0.67, 0.85]	0.71 [0.60, 0.80]
VO + HGS (n = 124)	0.74 [0.66, 0.81]	0.72 [0.63, 0.80]	0.75 [0.66, 0.83]	0.69 [0.61, 0.78]
**Class: VO**				
**Anterior Circulation**				
LVO (n = 41)	-	0.83 [0.70, 0.94]	0.88 [0.78, 0.96]	0.83 [0.70, 0.94]
MeVO (n = 29)	-	0.69 [0.50, 0.86]	0.72 [0.55, 0.88]	0.66 [0.48, 0.83]
**Posterior Circulation**				
VO (n = 19)	-	0.58 [0.33, 0.82]	0.58 [0.35, 0.83]	0.53 [0.29, 0.78]
**Class: VO + HGS**				
**Anterior Circulation**				
LVO (n = 49)	-	0.83 [0.73, 0.93]	0.88 [0.80, 0.95]	0.82 [0.71, 0.91]
MeVO (n = 29)	-	0.72 [0.57, 0.86]	0.75 [0.60, 0.89]	0.69 [0.54, 0.84]
**Posterior Circulation**				
VO (n = 19)	-	0.46 [0.27, 0.68]	0.46 [0.27, 0.68]	0.43 [0.24, 0.64]

Supplementary Table 12: Performance benchmarking of a CE-marked and FDA-cleared commercial software (blinded) in the FAST cohort, compared against HD-CTA. Comparisons were performed visually for all patients by considering only the detection of occlusions of the anterior circulation. Findings were considered correct as long as labelled on the correct vessel, in order to provide a fair comparison between the software. Findings labelled in vascular territories not considered by the commercial software (e.g. posterior circulation) were ignored. McNemar´s two-tailed test was used to compare specificity and sensitivity; comparison of relative predictive values was used instead to compare PPV and NPV (rpv.test function of R´s DTComPair package, two-tailed). P-values considered significant are highlighted in bold.

**FAST DATASET**						
**Software**	**AUROC**	**Accuracy**	**Sensitivity**	**Specificity**	**PPV**	**NPV**
**Overall [Occlusions n=39/320]**						
**HD-CTA**	0.89 [0.84-0.93]	0.86 [0.81-0.90]	0.92 [0.79-0.98]	0.85 [0.80-0.89]	0.46 [0.35-0.58]	0.99 [0.96-1.00]
Commercial Software 2	0.74 [0.66-0.82]	0.80 [0.75-0.84]	0.67 [0.50-0.81]	0.82 [0.77-0.86]	0.34 [0.23-0.45]	0.95 [0.91-0.97]
p-value	-	-	**p=0.003**	p=0.298	**p=0.021**	**p=0.003**
**LVO only [ICA, M1 – n=26]**						
**HD-CTA**	0.89 [0.83-0.94]	0.86 [0.81-0.89]	0.92 [0.75-0.99]	0.85 [0.80-0.89]	0.36 [0.25-0.49]	0.99 [0.97-1.00]
Commercial Software 2	0.83 [0.76-0.91]	0.82 [0.77-0.86]	0.85 [0.65-0.96]	0.82 [0.77-0.86]	0.30 [0.20-0.42]	0.98 [0.96-0.97]
p-value	-	-	p=0.321	p=0.30	p=0.174	p=0.296
**MeVO only [M2, M3 – n=13]**						
**HD-CTA**	0.89 [0.81-0.97]	0.85 [0.81-0.89]	0.92 [0.64-1.00]	0.85 [0.80-0.89]	0.22 [0.12-0.36]	1.00 [0.98-1.00]
Commercial Software 2	0.56 [0.43-0.70]	0.80 [0.75-0.84]	0.31 [0.09-0.61]	0.82 [0.77-0.86]	0.07 [0.02-0.18]	0.96 [0.93-0.98]
p-value	-	-	**p=0.004**	p=0.298	**p=0.006**	**p=0.004**

Supplementary Table 13· Number of false positive findings for each CTA acquisition phase within the FAST dataset. Pearson's chi-squared test was used for comparing the distribution of false positives. Distribution of false positives varies significantly across contrast phases both in the FAST and UKB dataset (p<0.001). P-values considered significant are highlighted in bold.

	**Acquisition phase within the FAST and UKB datasets**					
	**Early Arterial**	**Peak Arterial**	**Equilibrium**	**Peak Venous**	**Late Venous**	**p-value**
**FAST Dataset**						
**FP [n=48] (Object-level)**						
**False Positives**	3/48 (6%)	18/48 (38%)	25/48 (52%)	2/48 (4%)	0/48 (0%)	**p<0·001**
**False positives on veins [n=26]**	0/26 (0%)	11/26 (42%)	14/26 (54%)	1/26 (4%)	0/26 (0%)	**p<0·001**
**UKB Dataset**						
**FP [n=44] (Object-level)**						
**False Positives**	1/44 (2%)	6/44 (14%)	17/44 (39%)	12/44 (27%)	7/44 (16%)	**p<0·001**
**False positives on veins [n=27]**	1/27 (4%)	3/27 (11%)	10/27 (37%)	8/27 (30%)	4/27 (15%)	**p<0·001**
