# Supplementary material for: Deep-learning based detection of vessel occlusions on CT-angiography in patients with suspected acute ischemic stroke
Source: Nat Commun. 2023 Aug 15;14:4938. doi: 10.1038/s41467-023-40564-8 (PMC10427649; doi:10.1038/s41467-023-40564-8)
Supplement: Supplementary file 2 — Reporting Summary [file 41467_2023_40564_MOESM2_ESM.pdf]

nature portfolio

Corresponding author(s): Philipp Vollmuth
Last updated by author(s): Jan 13, 2023

Reporting Summary

Nature Portfolio wishes to improve the reproducibility of the work that we publish. This form provides structure for consistency and transparency in reporting. For further information on Nature Portfolio policies, see our Editorial Policies and the Editorial Policy Checklist.

Statistics

For all statistical analyses, confirm that the following items are present in the figure legend, table legend, main text, or Methods section.

n/a	Confirmed	
☐	☒	The exact sample size (*n*) for each experimental group/condition, given as a discrete number and unit of measurement
☐	☒	A statement on whether measurements were taken from distinct samples or whether the same sample was measured repeatedly
☐	☒	The statistical test(s) used AND whether they are one- or two-sided<br>*Only common tests should be described solely by name; describe more complex techniques in the Methods section.*
☐	☒	A description of all covariates tested
☐	☒	A description of any assumptions or corrections, such as tests of normality and adjustment for multiple comparisons
☐	☒	A full description of the statistical parameters including central tendency (e.g. means) or other basic estimates (e.g. regression coefficient) AND variation (e.g. standard deviation) or associated estimates of uncertainty (e.g. confidence intervals)
☐	☒	For null hypothesis testing, the test statistic (e.g. *F*, *t*, *r*) with confidence intervals, effect sizes, degrees of freedom and *P* value noted<br>*Give P values as exact values whenever suitable.*
☒	☐	For Bayesian analysis, information on the choice of priors and Markov chain Monte Carlo settings
☒	☐	For hierarchical and complex designs, identification of the appropriate level for tests and full reporting of outcomes
☐	☒	Estimates of effect sizes (e.g. Cohen's *d*, Pearson's *r*), indicating how they were calculated

Our web collection on statistics for biologists contains articles on many of the points above.

Software and code

Policy information about availability of computer code

Data collection	ADIT 0.23.3 (https://github.com/radexperts/adit), dcm2niix 1.0.2 (https://github.com/rordenlab/dcm2niix),
Data analysis	ITKsnap 3.8.0 (http://www.itksnap.org/), R 4.0.3, nnDetection 0.1 (https://github.com/MIC-DKFZ/nnDetection), DTComPair v. 1.2.0.

For manuscripts utilizing custom algorithms or software that are central to the research but not yet described in published literature, software must be made available to editors and reviewers. We strongly encourage code deposition in a community repository (e.g. GitHub). See the Nature Portfolio guidelines for submitting code & software for further information.

Data

Policy information about availability of data

All manuscripts must include a data availability statement. This statement should provide the following information, where applicable:

- Accession codes, unique identifiers, or web links for publicly available datasets
- A description of any restrictions on data availability
- For clinical datasets or third party data, please ensure that the statement adheres to our policy

The imaging data used for the study are protected and are not available due to data privacy laws. The de-identified data tables generated during and/or analysed during the current study are provided as source data file; any further de-identified data table are available from the corresponding author on reasonable request. The names of the two commercial software cannot be disclosed at any given point. Proposals and requests for data access should be directed to the corresponding author via email and will be responded to within the next 12 weeks.The development of the ANN is based on our previously published and publicly available nnDetection method available via https://github.com/MIC-DKFZ/nnDetection. The authors will be available at any time to apply the developed ANN onto external

data provided by other academic researchers on their behalf (for research purposes only), following completion of a Material Transfer Agreement. Proposals and requests for data access should be directed to the corresponding author via email. A user-friendly end-to-end workflow of the developed ANN is publicly available (for research purposes only) through https://stroke.neuroAI-HD.org.

Human research participants

Policy information about studies involving human research participants and Sex and Gender in Research.

Reporting on sex and gender	Data Collected and available in Data Supplement - Table 2. The research findings apply to both male and female sex, as both populations are included in the training and testing cohorts. The distribution of male:female is reported in Supplementary Table 2 and shows non-significant differences across all datasets.
Population characteristics	Demographic data on the patient population is listed in the Data Supplement - Table 2.
Recruitment	The Heidelberg cohort included 800 consecutive patients with AIS and confirmed vessel occlusion on CT-angiography who subsequently underwent EVT between 03/2010 and 02/2020, as well as 379 consecutive patients with a suspected diagnosis of stroke but no vessel occlusion (control group) who underwent CT-angiography between 10/2019 and 02/2020. Pseudo-prospective external testing of the ANN was performed onto two different datasets, and namely (i) the FAST cohort, with 358 consecutive patients who underwent CT-angiography between 01/2022 and 06/2022 for suspected AIS at three primary/secondary care hospitals of the regional stroke consortium Rhine-Neckar with acute teleneurology/teleradiology coverage through the Heidelberg University Hospital, and the UKB cohort, with 323 patients who underwent CT-angiography between 09/2020 and 04/2021 for suspected AIS at the Department of Neuroradiology of the Bonn University Hospital.
Ethics oversight	The study was approved by the ethics committee of the University of Heidelberg

Note that full information on the approval of the study protocol must also be provided in the manuscript.

Field-specific reporting

Please select the one below that is the best fit for your research. If you are not sure, read the appropriate sections before making your selection.

[x] Life sciences [ ] Behavioural & social sciences [ ] Ecological, evolutionary & environmental sciences

For a reference copy of the document with all sections, see nature.com/documents/nr-reporting-summary-flat.pdf

Life sciences study design

All studies must disclose on these points even when the disclosure is negative.

Sample size	The internal sample size was determined by the availability of data in our records (all data available was included). The external data samples were collected by setting a fixed time period of six months and collecting all available data within said timeframe.
Data exclusions	See Figure 1 in the Main manuscript body for a list of the patient exclusions from the data samples. Patients were excluded mainly based insufficient on data quality or data corruption.
Replication	After testing our tool on the internal sample, we used two external data cohorts to reproduce our findings. The ANN showed consistent results also in the external testing samples.
Randomization	Not relevant to this study, no randomization was performed. The study is retrospective in nature and the same treatment was applied to the entire sample in all cases.
Blinding	Not relevant to this study, no randomization was performed. The study is retrospective in nature and the same treatment was applied to the entire sample in all cases.

Reporting for specific materials, systems and methods

We require information from authors about some types of materials, experimental systems and methods used in many studies. Here, indicate whether each material, system or method listed is relevant to your study. If you are not sure if a list item applies to your research, read the appropriate section before selecting a response.

Materials & experimental systems

n/a	Involved in the study
☒	☐ Antibodies
☒	☐ Eukaryotic cell lines
☒	☐ Palaeontology and archaeology
☒	☐ Animals and other organisms
☒	☐ Clinical data
☒	☐ Dual use research of concern

Methods

n/a	Involved in the study
☒	☐ ChIP-seq
☒	☐ Flow cytometry
☒	☐ MRI-based neuroimaging
